# Correction: Selection on a Variant Associated with Improved Viral Clearance Drives Local, Adaptive Pseudogenization of Interferon Lambda 4 (*IFNL4*)

**DOI:** 10.1371/journal.pgen.1004882

**Published:** 2014-11-19

**Authors:** The PLOS Genetics Staff

## Notice of Republication

This article was republished on November 7, 2014, to correct errors in Figure 3 and the Author Contributions that were introduced during the preparation of the manuscript. Please download this article again to view the correct version. The originally published, uncorrected article and the republished, corrected article are provided here for reference.

## Supporting Information

File S1 Originally published, uncorrected article.
(PDF)

File S2 Republished corrected article.
(PDF)
