# Supplementary material for: Selection on a Variant Associated with Improved Viral Clearance Drives Local, Adaptive Pseudogenization of Interferon Lambda 4 (IFNL4)
Source: PLoS Genet. 2014 Oct 16;10(10):e1004681. doi: 10.1371/journal.pgen.1004681 (PMC4199494; doi:10.1371/journal.pgen.1004681)
Supplement: Figure S1 — Empirical P-values of the FST and XP-EHH analyses for all non-Asian. populations using YRI as background within a +−15 kb region around rs368234815. (PDF) [file pgen.1004681.s001.pdf]

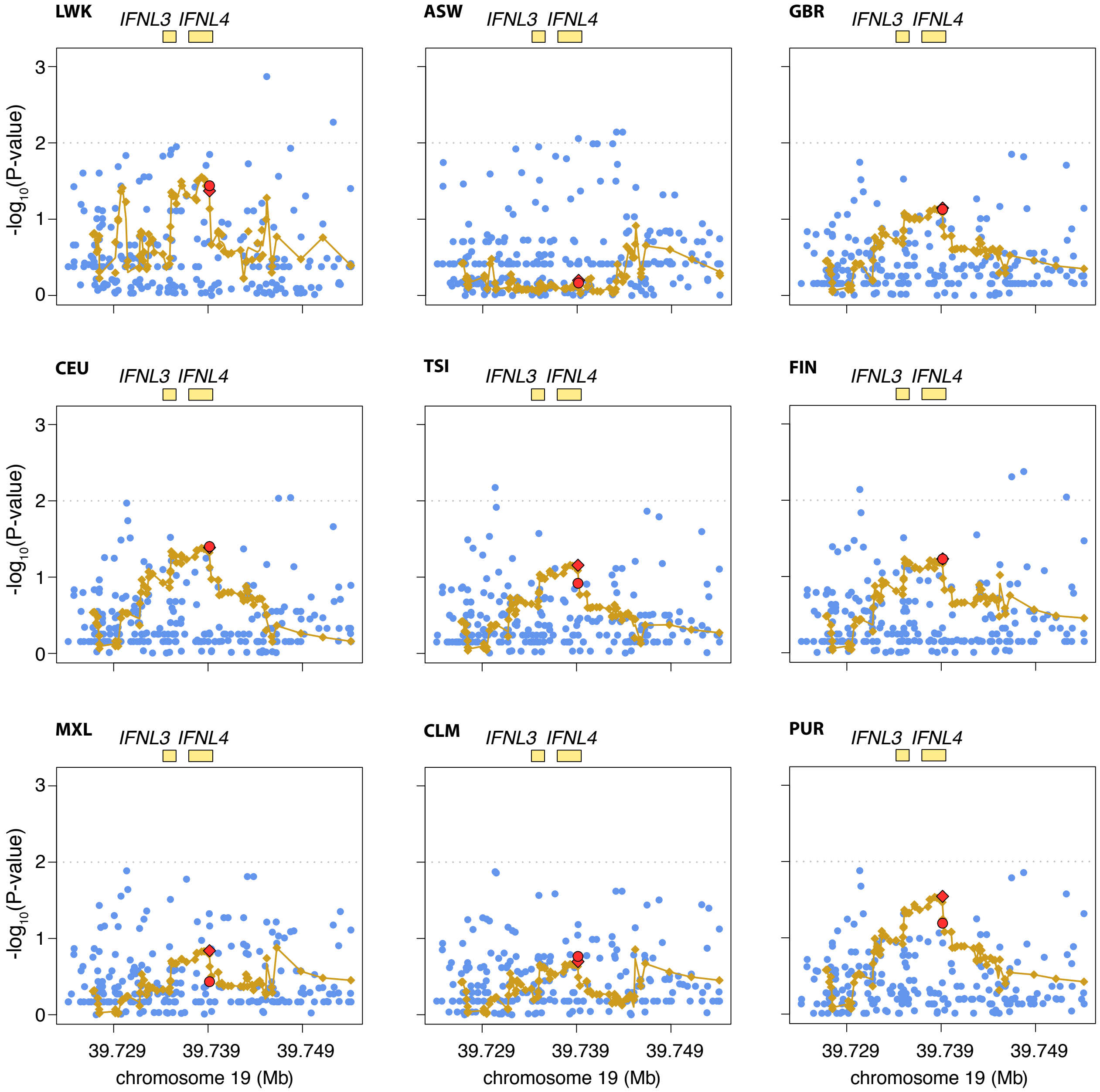

Supplementary Figure 1. Empirical P-values of the F_{ST} and XP-EHH analyses for all non-Asian populations using YRI as background within a +-15kb region around rs368234815.

●/◆ denotes F_{ST} and XP-EHH for rs368234815 respectively; ●/◆ denotes F_{ST} and XP-EHH for all other variants in the region. All XP-EHH values are connected by a fitting curve (yellow line). The 1% tail of the genomic empirical distribution is indicated by the horizontal, dashed line.
