# Supplementary material for: Selection on a Variant Associated with Improved Viral Clearance Drives Local, Adaptive Pseudogenization of Interferon Lambda 4 (IFNL4)
Source: PLoS Genet. 2014 Oct 16;10(10):e1004681. doi: 10.1371/journal.pgen.1004681 (PMC4199494; doi:10.1371/journal.pgen.1004681)
Supplement: Figure S2 — Empirical P-values of the XP-EHH and FST analysis in the IFNL cluster for all populations. (PDF) [file pgen.1004681.s002.pdf]

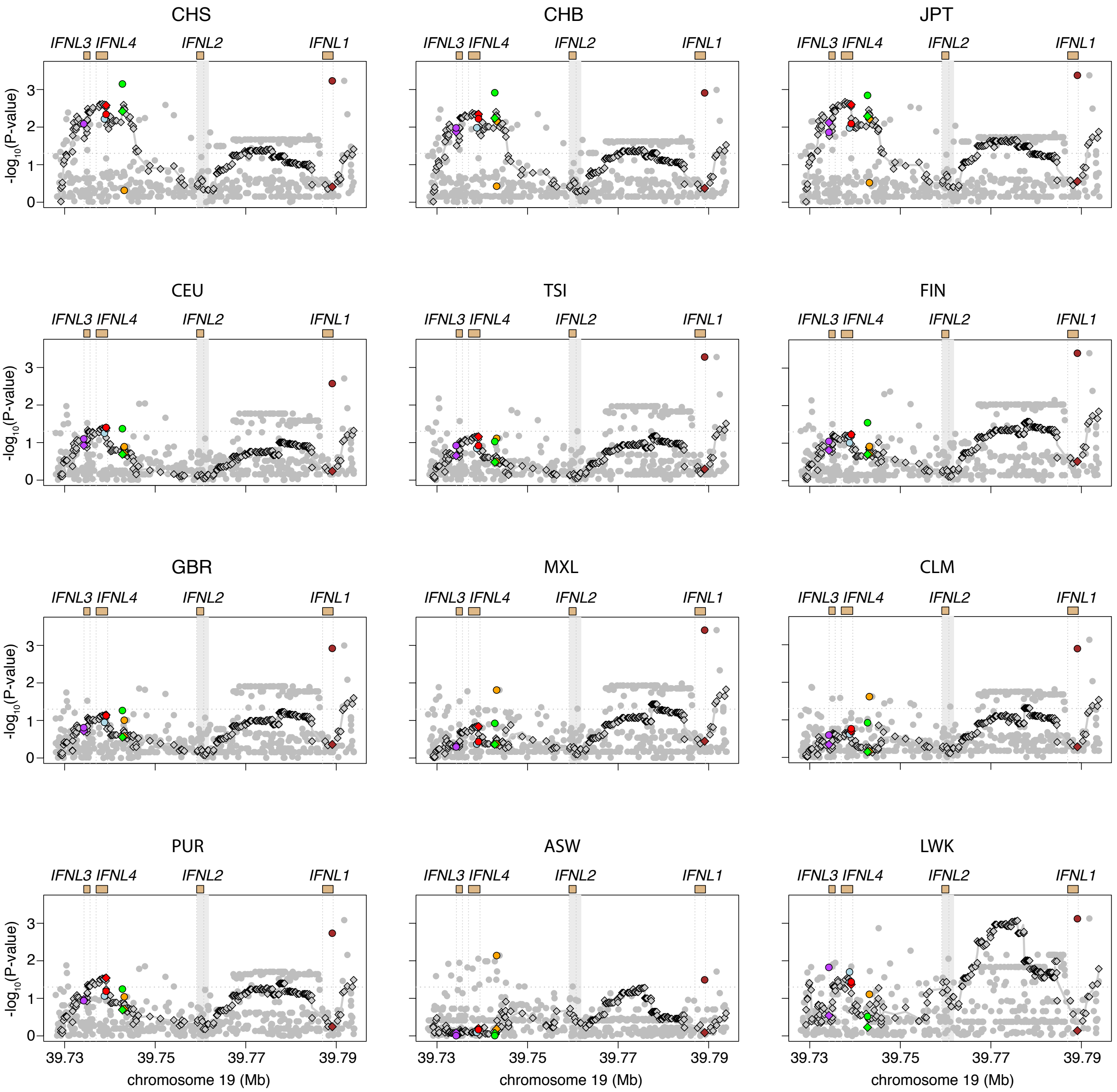

Supplementary Figure 2. Empirical P-values of the XP-EHH and F_{ST} analysis in the *IFNL* cluster for all populations.

Diamonds/Dots indicate the results for XP-EHH and F_{ST} respectively. ◆/● rs368234815; ◇/○ rs12979860; ◆/● rs8099917; ◆/● rs8109886; ◆/● rs30461; ◆/● rs4803217; ◇/● all other variants in the *IFNL* cluster. All XP-EHH values are connected by a gray fitting curve. The horizontal dashed line indicates the 5% tail of the empirical distribution and the grey shaded area the recombination hotspot.
