# Supplementary material for: Selection on a Variant Associated with Improved Viral Clearance Drives Local, Adaptive Pseudogenization of Interferon Lambda 4 (IFNL4)
Source: PLoS Genet. 2014 Oct 16;10(10):e1004681. doi: 10.1371/journal.pgen.1004681 (PMC4199494; doi:10.1371/journal.pgen.1004681)
Supplement: Figure S3 — Haplotype structure +−15 Kb around rs368234815 for three population per continent. (PDF) [file pgen.1004681.s003.pdf]

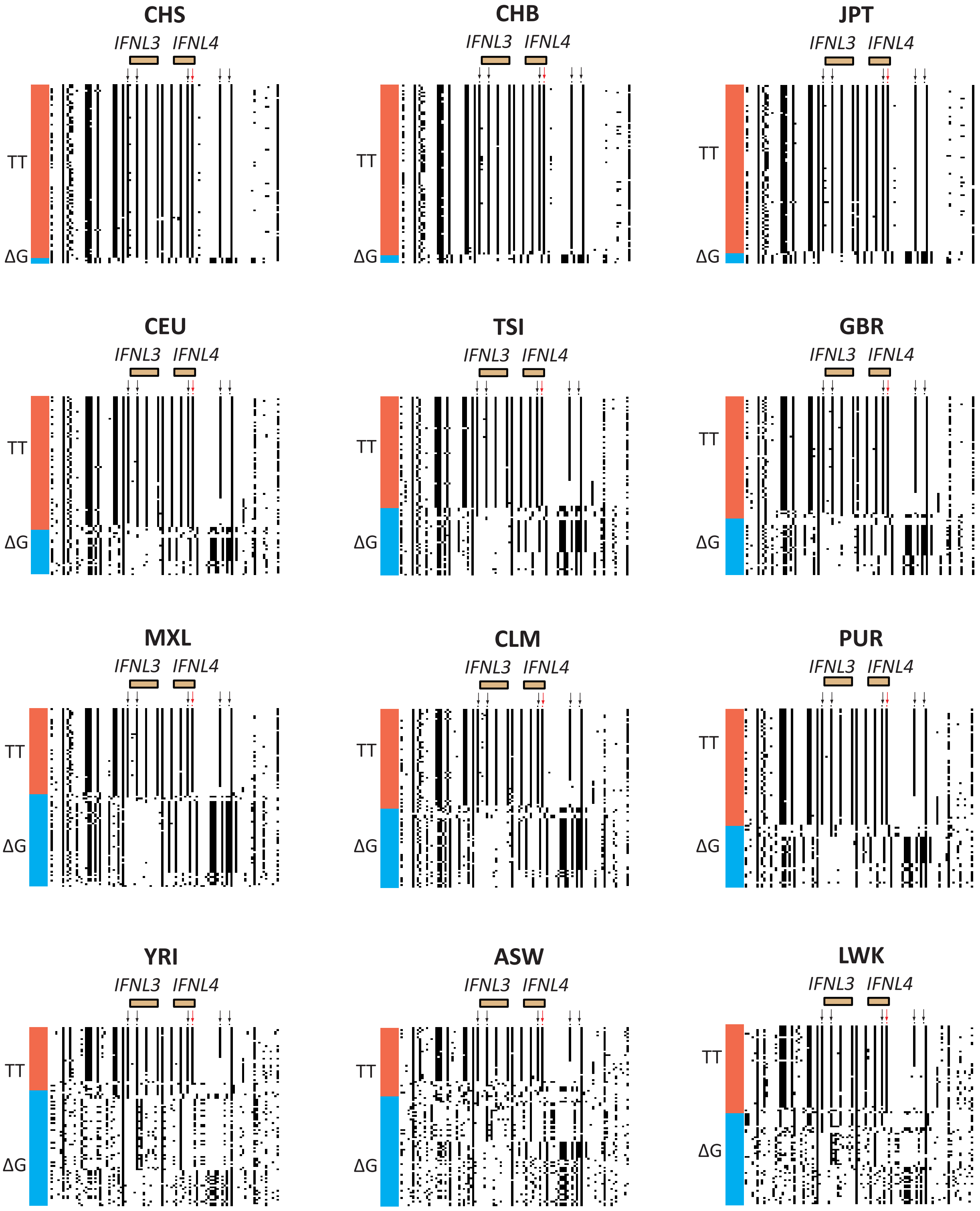

Supplementary Figure 3. Haplotype structure +-15Kb around rs368234815 for three population per continent.

Columns represent SNPs with a derived allele frequency > 5% in at least one population (n=99 SNPs), with the ancestral allele in white, and the derived allele in black. Horizontal lines represent the haplotypes they fall in, as inferred with SHAPEIT by the 1000 Genomes consortium {Mcvean 2012}. Haplotypes were sorted based on rs368234815 (red arrow) and SNPs in perfect LD with it in CHS (black arrows); see also Table 2 and Figure 4. The bar on the left-hand side of each plot indicates haplotypes carrying the TT allele (red) or the ΔG allele (blue).
