# Supplementary material for: Selection on a Variant Associated with Improved Viral Clearance Drives Local, Adaptive Pseudogenization of Interferon Lambda 4 (IFNL4)
Source: PLoS Genet. 2014 Oct 16;10(10):e1004681. doi: 10.1371/journal.pgen.1004681 (PMC4199494; doi:10.1371/journal.pgen.1004681)
Supplement: Figure S4 — Haplotype network for IFNL4. (PDF) [file pgen.1004681.s004.pdf]

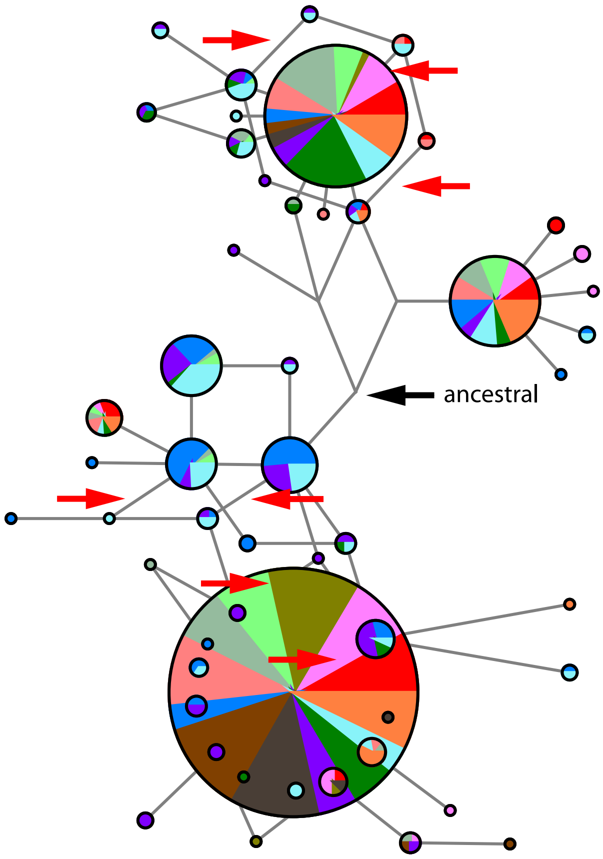

Supplementary Figure 4. Shown is the haplotype network of *IFNL4* (incl. UTRs). Circles represent haplotypes with the areas proportional to the frequency of the haplotype and color-coded by population (YRI:●; ASW:●; LWK:●; CEU:●; GBR:●; TSI:●; FIN:●; PUR:●; MXL:●; CLM:●; JPT:●; CHB:●; CHS:●). The lines connecting the haplotypes have a length proportional to the number of mutations that differentiate the two haplotypes. The TT mutation is indicated by a red arrow. Reticulations reflect recombinations or recurrent mutations. The ancestral state was inferred using chimpanzee (UCSC, pantro3).
