# Supplementary material for: Selection on a Variant Associated with Improved Viral Clearance Drives Local, Adaptive Pseudogenization of Interferon Lambda 4 (IFNL4)
Source: PLoS Genet. 2014 Oct 16;10(10):e1004681. doi: 10.1371/journal.pgen.1004681 (PMC4199494; doi:10.1371/journal.pgen.1004681)
Supplement: Figure S6 — Parameter estimates for the supra-additive model using a (a) SDN or (b) SSV model of selection for Asian and European populations. (PDF) [file pgen.1004681.s006.pdf]

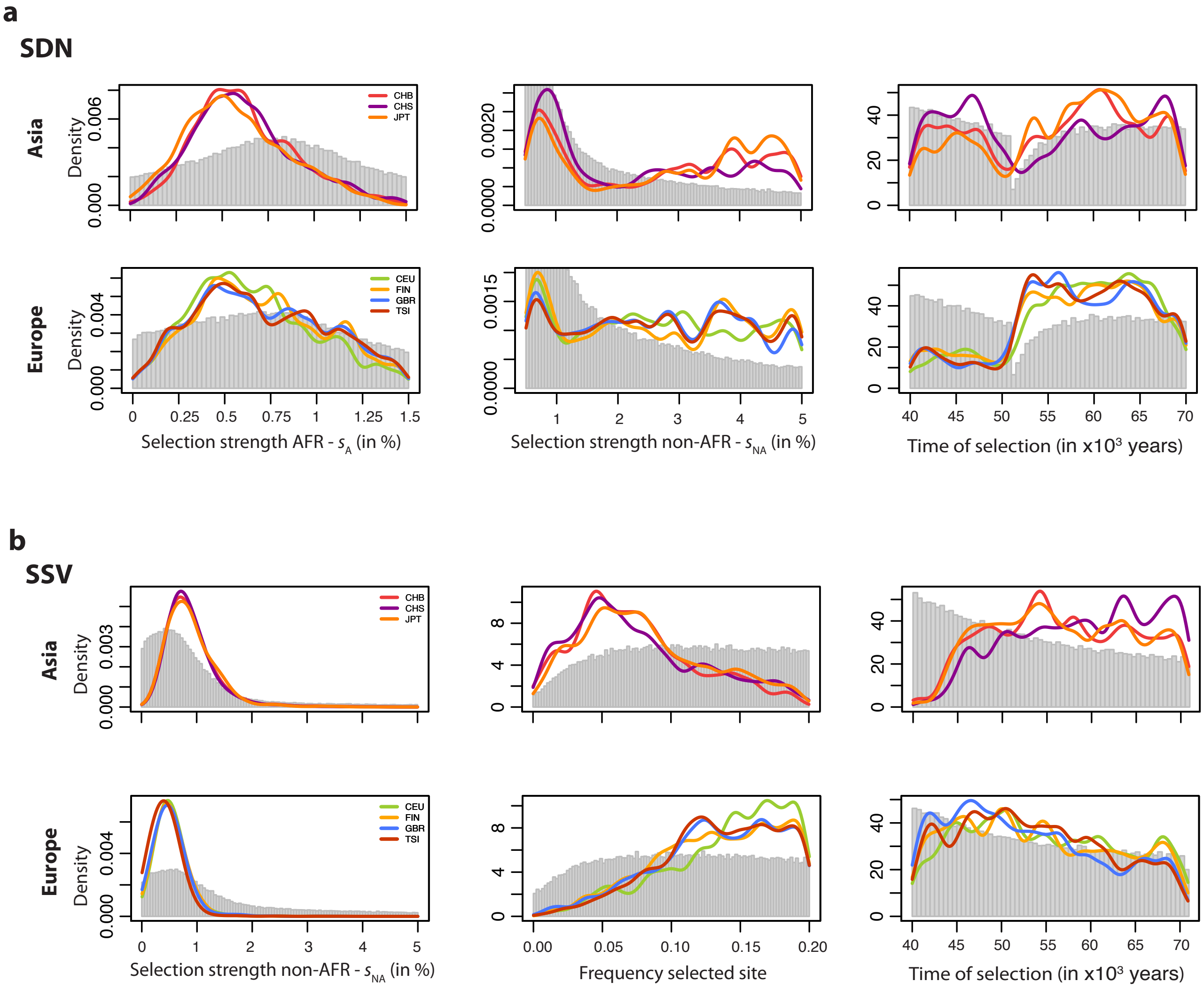

Supplementary Figure 6. Parameter estimates for the supra-additive model using a (a) SDN or (b) SSV selection model for Asian and European populations. The histogram in gray indicates the prior distribution and the colored lines the posterior estimate for each population.
