# Supplementary material for: Selection on a Variant Associated with Improved Viral Clearance Drives Local, Adaptive Pseudogenization of Interferon Lambda 4 (IFNL4)
Source: PLoS Genet. 2014 Oct 16;10(10):e1004681. doi: 10.1371/journal.pgen.1004681 (PMC4199494; doi:10.1371/journal.pgen.1004681)
Supplement: Figure S7 — Linkage disequilibrium (LD) patterns across the IFNL locus in three representative populations ((a) YRI, (b) CEU, (c) CHB). (PDF) [file pgen.1004681.s007.pdf]

a) YRI

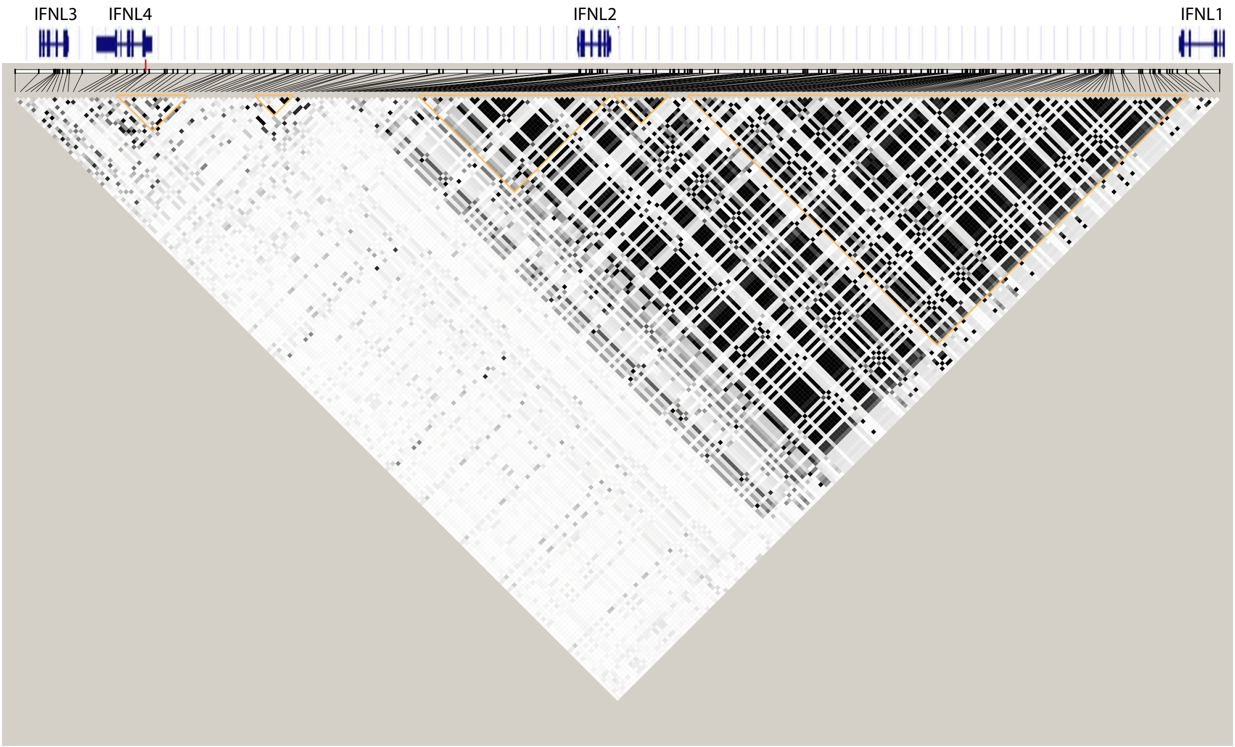

b) CEU

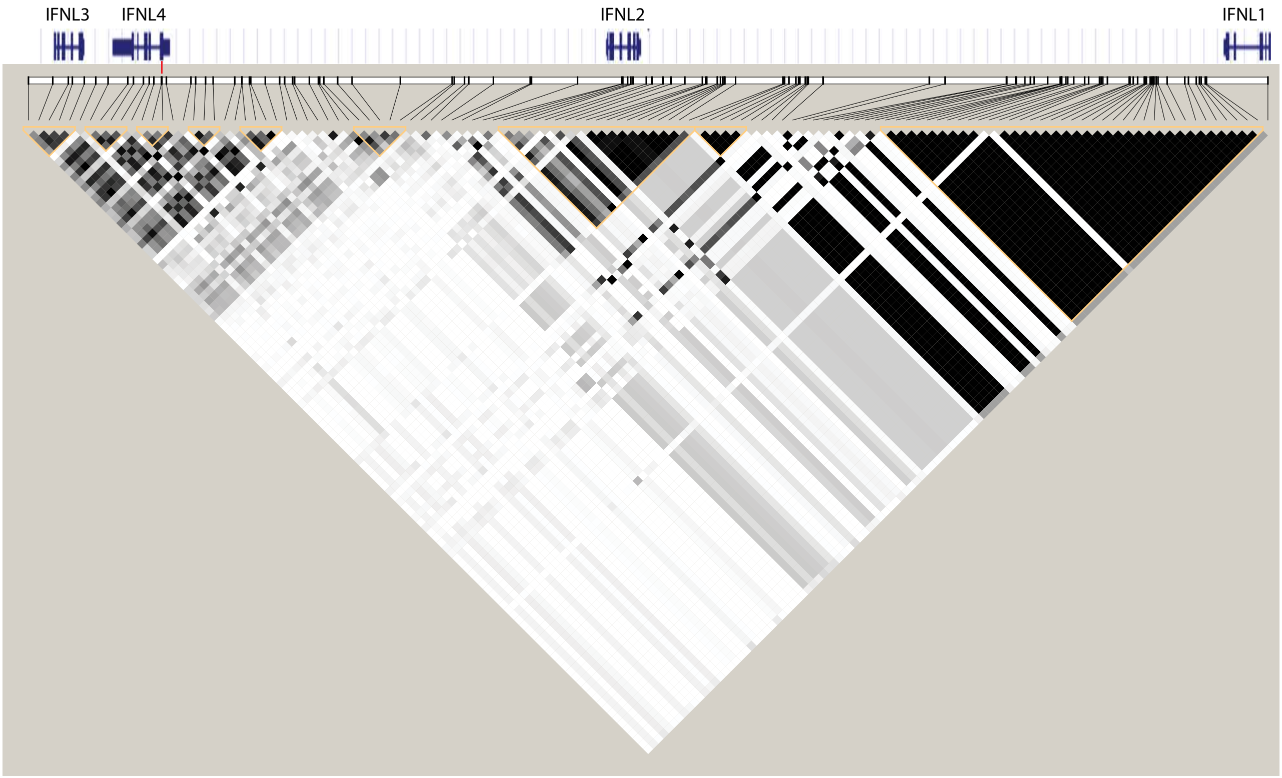

c) CHB

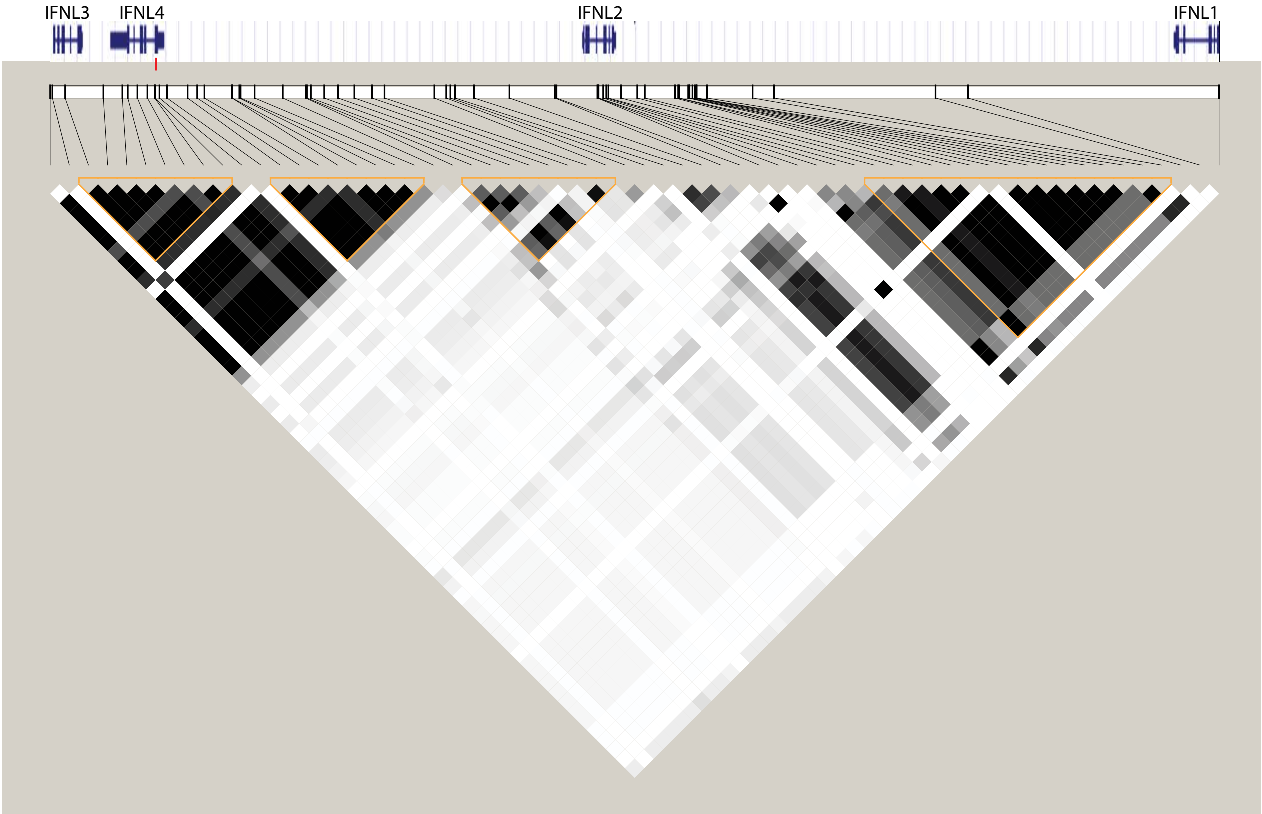

Supplementary Figure 7. Linkage disequilibrium (LD) patterns across the *IFNL* locus in three representative populations (**a**) YRI, (**b**) CEU, (**c**) CHB) using 100 chromosomes per population. LD is estimated using r2 and assessment of LD blocks (yellow lines) follows Gabriel et. al [1]. The red line indicates the position of rs368234815.

1. Gabriel SB, Schaffner SF, Nguyen H, Moore JM, Roy J, et al (2002) The Structure of Haplotype Blocks in the Human Genome. Science 296: 2225-2229.
