# Supplementary material for: Selection on a Variant Associated with Improved Viral Clearance Drives Local, Adaptive Pseudogenization of Interferon Lambda 4 (IFNL4)
Source: PLoS Genet. 2014 Oct 16;10(10):e1004681. doi: 10.1371/journal.pgen.1004681 (PMC4199494; doi:10.1371/journal.pgen.1004681)
Supplement: Note S1 — Assessment of selection on other variants in the IFNL-locus. (PDF) [file pgen.1004681.s008.pdf]

Supplementary Note 1. Assessment of selection on other variants in the *IFNL* locus

A classical problem in population genetics is the identification of the particular genetic variant responsible for selection signals. The rs368234815 TT allele has a strong phenotypic effect by truncating *IFNL4*, but we cannot *per se* rule out that another allele on the TT haplotype drives the selective signature. As **Figure 3a-c** and **Supplementary Figure 2** show there are other variants with comparable F_{ST} or XP-EHH values. The two variants initially described as being associated with HCV clearance are rs12979860 [1,2] and rs8099917 [3]. Both variants show weaker combined selection signals than the TT allele (**Supplementary Fig. 2**). This is in line with the unclear functional effects of these variants [4-8] and their weaker association with HCV clearance compared to rs368234815 in several populations [9-11]. Thus, rs12979860 and rs8099917 are much weaker candidates to be the target of natural selection.

Only one variant (rs8109886, A>C, 19:39742762), which is intergenic and located 3.3Kb upstream of *IFNL4*, also showed the combined signatures of positive selection (XP-EHH and iHS) and high population differentiation (**Table X1**, **Supplementary Fig. 2**). While the rs368234815 TT allele has a very clear functional consequence, no functional effect has been described for rs8109886. Selection can act only on variants with functional and phenotypic consequences, making *a priori* the TT allele a much more reasonable candidate for selection. Still, to tease apart the signatures of the two variants, we analyzed the frequencies of the four possible allele configurations (haplotypes) of both variants (**Figure X1**). The **Figure X1** shows that the derived C allele of rs8109886 resides almost exclusively on the TT haplotype (a single ΔG-C haplotype in LWK is likely the result of recombination), suggesting the derived C allele appeared in the TT background. **Figure X1** also shows a change of rs8109886 allele frequencies on the TT background: while both rs8109886 alleles are equally frequent in Africa, TT-C haplotype has a higher frequency than TT-A haplotype in all other populations. To determine whether this observation is compatible with a model of selection on TT alone we used simulations with the parameters for the SDN model inferred in the ABC approach (see **Methods**, **SI Note 3**, **Table 3**). Given the frequencies measured for TT in Africa and Asia, and for C in Africa, we obtained a probability of 23.8% for the C variant to reach similar or higher frequency in Asia than that we observed. Thus, the skewed pattern of the TT-C haplotype in Asia, and the higher F_{ST} for C, are not unexpected by selection on TT alone. While we cannot completely discard the possibility that C alone has been under positive selection, and *IFNL4* disruption by TT is a byproduct of selection on this linked variant, this is a very unlikely scenario because: (1) No function has been observed for rs8109886 despite being extensively scrutinized due to the association of this genomic region with HCV clearance; (2) the TT variant has a clear and important functional consequence; (3) TT has in addition a very strong signature of positive selection; and (4) selection favors an allele that shows an advantage against specific viral infections. In any case, several lines of evidence indicate that the high frequency of IFN-λ4 pseudogenization through disruption by the TT allele is the consequence of positive selection in East Asia.

Another variant, rs4803217 (within IFNL3 3‘UTR), was recently shown to be functional and thus suggested to contribute to the HCV association in this region [12]. However, this variant shows weaker selection signals than rs368234815, most notably in European populations where LD

between these variants is less strong than in Asians, and in African populations, where these variants are only in moderate LD (r^2= 0.62, **Table 2, Fig. S2**).

There is moderate LD between *IFNL4* and *IFNL3*, with an average r^2 between rs368234815 and SNPs in *IFNL3* of 0.18 in CEU and of 0.44 in CHS (see also Fig. S7). So, the selection signatures in *IFNL3* and *IFNL4* may not be independent. In fact, the four *IFNL3* SNPs (rs11881222, rs12979860, rs8109886, rs8099917) identified by Manry et al. [13] are in high LD with rs368234815 but have (1) unclear functional effects, (2) weaker association with HCV clearance than the TT allele at least in Africa, and, importantly, (3) weaker signatures of selection (**Table 2**). Thus, the signatures for these variants are most likely driven by linkage with rs368234815, which is under positive selection (**Fig. S2, Table 2**). Some interesting variants (rs8103142, rs28416813) which also have high derived allele frequencies (DAF) in CHS and signatures of selection show moderate LD with rs368234815 (**Table 2**). Still, the signatures for these variants are most likely not independent from rs368234815 because LD is reduced by recombination events that likely affect the ancestral haplotype. Of the 47 individuals homozygous for the TT allele in CHS, all are also homozygous for the derived allele of rs8103142, and 46 are also homozygous for the derived allele of rs28416813. Taken together, these lines of evidence confirm that *IFNL1/2* and *IFNL3/IFNL4* have likely been independently targeted by positive selection in recent human history, as suggested by Manry et al. [13], and highlight that the TT allele of rs368234815 is the most likely selected allele in this region.

Table X1. Analyses of rs8109886. Derived allele frequency, linkage disequilibrium (r^2) with rs368234815, and empirical P-values for F_{ST}, XP-EHH, and iHS based on a whole-genome analysis for every population. F_{ST} and XP-EHH use YRI as background population.

Population	Frequency	r^2	p(F_{ST})	p(XP)	p(iHS)
CHS	0.97	1	0.001	0.004	NA*
CHB	0.94	0.8	0.001	0.006	0.07
JPT	0.93	1	0.001	0.005	0.036
GBR	0.55	0.6	0.054	0.279	0.014
CEU	0.58	0.5	0.043	0.205	0.018
FIN	0.63	0.7	0.028	0.199	0.031
TSI	0.47	0.6	0.093	0.333	0.028
CLM	0.42	0.6	0.119	0.711	0.016
MXL	0.44	0.9	0.119	0.434	0.364
PUR	0.55	0.5	0.056	0.202	0.052
ASW	0.19	0.4	0.979	0.86	0.262
LWK	0.25	0.3	0.302	0.592	0.999
YRI	0.17	0.4	-$	-$	0.636

* iHS could not be computed for rs368234815 because the allele frequency is too high.

$ YRI is used as background population for that analysis

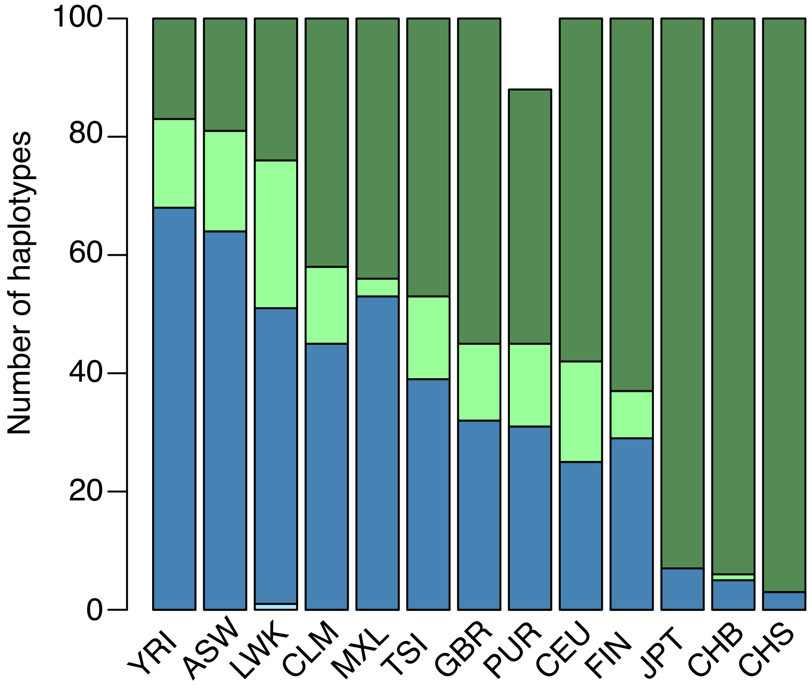

Figure X1. Frequency of the different haplotypes of rs368234815 and rs8109886 in each population. Haplotypes are denoted as followed: ΔG-A; ΔG-C; TT-A; and TT-C.

References

1. Ge D, Fellay J, Thompson AJ, Simon JS, Shianna KV, et al (2009) Genetic variation in IL28B predicts hepatitis C treatment-induced viral clearance. Nature 461: 399-401.
2. Thomas DL, Thio CL, Martin MP, Qi Y, Ge D, et al (2009) Genetic variation in IL28B and spontaneous clearance of hepatitis C virus. Nature 461: 798-801.
3. Suppiah V, Moldovan M, Ahlenstiel G, Berg T, Weltman M, et al (2009) IL28B is associated with response to chronic hepatitis C interferon-α and ribavirin therapy. Nat Genet 41: 1100-1104.
4. Dill MT, Duong FH, Vogt JE, Bibert S, Bochud P, et al (2011) Interferon-Induced Gene Expression Is a Stronger Predictor of Treatment Response Than {IL28B} Genotype in Patients With Hepatitis C. Gastroenterology 140: 1021 - 1031.e10.
5. Fukuhara T, Taketomi A, Motomura T, Okano S, Ninomiya A, et al (2010) Variants in {IL28B} in Liver Recipients and Donors Correlate With Response to Peg-Interferon and Ribavirin Therapy for Recurrent Hepatitis C. Gastroenterology 139: 1577 - 1585.e3.
6. Langhans B, Kupfer B, Braunschweiger I, Arndt S, Schulte W, et al (2011) Interferon-lambda serum levels in hepatitis C. J Hepatol 54: 859 - 865.
7. Honda M, Sakai A, Yamashita T, Nakamoto Y, Mizukoshi E, et al (2010) Hepatic {ISG} Expression Is Associated With Genetic Variation in Interleukin 28B and the Outcome of {IFN} Therapy for Chronic Hepatitis C. Gastroenterology 139: 499 - 509.
8. Urban TJ, Thompson AJ, Bradrick SS, Fellay J, Schuppan D, et al (2010) IL28B genotype is associated with differential expression of intrahepatic interferon-stimulated genes in patients with chronic hepatitis C. Hepatology 52: 1888-1896.
9. Aka P, Kuniholm MH, Pfeiffer RM, Wang AS, Tang W, et al (2013) Association of the IFNL4-ΔG Allele with Impaired Spontaneous Clearance of Hepatitis C Virus. Journal of Infectious Diseases .
10. Bibert S, Roger T, Calandra T, Bochud M, Cerny A, et al (2013) IL28B expression depends on a novel TT/-G polymorphism which improves HCV clearance prediction. J Exp Med 210: 1109-1116.
11. Prokunina-Olsson L, Muchmore B, Tang W, Pfeiffer RM, Park H, et al (2013) A variant upstream of IFNL3 (IL28B) creating a new interferon gene IFNL4 is associated with impaired clearance of hepatitis C virus. Nat Genet 45: 164.
12. McFarland AP, Horner SM, Jarret A, Joslyn RC, Bindewald E, et al (2013) The favorable IFNL3 genotype escapes mRNA decay mediated by AU-rich elements and hepatitis C virus–induced microRNAs. Nat Immunol 15: 72-79.
13. Manry J, Laval G, Patin E, Fornarino S, Itan Y, et al (2011) Evolutionary genetic dissection of human interferons. J Exp Med 208: 2747-2759.
