# Supplementary material for: Selection on a Variant Associated with Improved Viral Clearance Drives Local, Adaptive Pseudogenization of Interferon Lambda 4 (IFNL4)
Source: PLoS Genet. 2014 Oct 16;10(10):e1004681. doi: 10.1371/journal.pgen.1004681 (PMC4199494; doi:10.1371/journal.pgen.1004681)
Supplement: Note S2 — Geographical origin of the rs368234815 TT variant. (PDF) [file pgen.1004681.s009.pdf]

Supplementary Note 2. Geographical origin of the rs368234815 TT allele

As the frequency of TT is higher outside of Africa than in Africa, we addressed the unlikely possibility that the variant appeared outside of Africa and subsequently migrated back to Africa. Diversity accumulated on the *IFNL4*-TT haplotype is expected to be proportional to the time the variant is present in a population, under the assumptions that the ΔG>TT mutation happened only once, and that nearby variants are largely neutral. As derived TT is a complex mutation and leads to inactivation of *IFNL4* both assumptions are reasonable.

To investigate the diversity in *IFNL4* on the derived TT background we used the following criteria; (1) we used only homozygous individuals TT/TT and (2) we compared our results to neutral regions. First, using only homozygous individuals excluded inaccurate phasing as a source of error. All populations had at least nine homozygous TT/TT individuals (which we randomly chose) except ASW that had only seven. Second, it is known that the level of diversity across human populations varies. To put our results in the context of neutral population diversity we also estimated diversity for a set of pseudogenes across the genome in the same individuals. Specifically, we used a set of pseudogenes recently described by GENCODE [1], which integrated functional genomic information from ENCODE [2]. We required every pseudogene to be autosomal and of retrotransposition origin, and to lack any indication of transcriptional activity or open chromatin state. This resulted in 867 pseudogenes throughout the human genome covering a total of ~560 Kb. As a measure of diversity we used the Watterson estimator [3], which is based on the number of segregating sites.

The highest associated diversity of TT haplotypes was observed in Africa (**Supplementary Table 5**), which is in line with genome-wide estimates of diversity [4] and points to an African origin for this variant. Specifically, we estimate the highest diversity for TT haplotypes and the control regions in LWK (Luhya, Kenya, East Africa). However, the diversity in LWK for the TT haplotypes in comparison to YRI (second most diverse; Yoruba, Nigeria, West Africa) is 25% higher while in the control regions this is merely 3%. Even though a chi-square test is not significant (P-value = 0.69) due to small sample size, this excess of diversity for LWK suggests that TT may have appeared in a population in Africa more closely related to the LWK population. Outside of Africa only one TT haplotype is present, which carries the TT allele and three linked sites (data not shown). In Africa, additional low-frequency variants accumulated on the TT haplotype. The physical position of these low-frequency variants is interspersed with the high-frequency variants in the locus. Thus the results indicate an African origin, most likely in a population that was closer to present-day East African populations such as LWK.

Another way to visualize the relationship among haplotypes in the different populations is a haplotype network. We created the haplotype network for the *IFNL4* region (incl. UTRs, chr19:39736954-39739496) for all populations using the software Network [5]. As outgroup we used chimpanzee (pantro3 lastz alignment from UCSC). **Figure X2** shows the resulting network for *IFNL4* region and reveals again that the largest variation is in the African populations. The biggest cluster carries the derived TT variant and contains individuals from every population. In addition,

haplotypes carrying the TT allele are present elsewhere, which can be explained by isolated recombination events.

References

1. Pei B, Sisu C, Frankish A, Howald C, Habegger L, et al (2012) The GENCODE pseudogene resource. Genome Biol 13: R51.

2. Dunham I, Kundaje A, Aldred SF, Collins PJ, Davis CA, et al (2012) An integrated encyclopedia of DNA elements in the human genome. Nature 489: 57-74.

3. Watterson GA (1975) On the number of segregating sites in genetical models without recombination. Theor Popul Biol 7: 256-276.

4. McVean GA, Altshuler (Co-Chair) DM, Durbin (Co-Chair) RM, Abecasis GR, Bentley DR, et al (2012) An integrated map of genetic variation from 1,092 human genomes. Nature 491: 56-65.

5. Bandelt HJ, Dress AWM (1992) Split decomposition: a new and useful approach to phylogenetic analysis of distance data. Mol Phylogenet Evol 1: 242-252.
