# Supplementary material for: Selection on a Variant Associated with Improved Viral Clearance Drives Local, Adaptive Pseudogenization of Interferon Lambda 4 (IFNL4)
Source: PLoS Genet. 2014 Oct 16;10(10):e1004681. doi: 10.1371/journal.pgen.1004681 (PMC4199494; doi:10.1371/journal.pgen.1004681)
Supplement: Note S3 — Performance of the ABC approach. (PDF) [file pgen.1004681.s010.pdf]

**Supplementary Note 3. Performance of the ABC approach**

As with any ABC approach, it is important to assess the accuracy and fit of the inference procedure to the data. To this end, we used 10,000 simulations from each model to test the performance of ABC for each of our models. In particular, we performed the following tests: i) we checked if we were able to fit the observed data with the simulations from the models we used; ii) we checked for bias and power in the model choice procedure we used; iii) we checked for bias and accuracy for parameter estimation under the favored SDN model.

<u>Model fit</u>

We first tested if our models are capable of simulating summary statistics similar to the ones we observed in the 1000 Genomes data. For simplicity, we will just report the best fitting models for one European (CEU) and one Asian (CHB) population. In **Tables X2** and **X3**, we contrast the summary statistics of the ten most closely fitting simulations for CEU and CHB respectively, along with the statistics calculated from the observed data. In **Figures X3** and **X4**, we show the fit of all pairwise transformed statistics, which were used for inference. Overall, we find that our simulations match the observed data reasonably well. Some of the theta estimators seem hard to match, in particularly θH and thus Fay and Wu's H are often different even for simulations that match the data well. For the transformed statistics (**Figures X3** and **X4**), we similarly find that all simulated data set lie within the range of the models.

**Table X2**. Summary statistics of observed data (first row) and best fitting simulations (rows 2-11) for the CHB data.

| SimNo. | θPi | θW | θH | TD | FWH | θPi2 | θW2 | θH2 | TD2 | FWH2 | fpop1 | fpop2 | $F_{ST}$ | $F_{ST}$-sel | $F_{ST}2$ | XPEHH |
|---|---|---|---|---|---|---|---|---|---|---|---|---|---|---|---|---|
| **CHB** | *1.083* | *1.93* | *7.26* | *-1.123* | *6.18* | *1.70* | *3.28* | *16.59* | *-1.37* | *14.89* | *32* | *95* | *0.225* | *0.596* | *0.313* | *-1.85* |
| **47719** | 1.329 | 2.12 | 9.74 | -0.977 | 8.41 | 1.43 | 2.90 | 9.74 | -1.40 | 8.31 | 29 | 91 | 0.221 | 0.568 | 0.425 | -1.70 |
| **73116** | 0.786 | 1.74 | 8.53 | -1.367 | 7.74 | 2.71 | 4.25 | 13.51 | -1.06 | 10.80 | 37 | 99 | 0.221 | 0.609 | 0.369 | -1.66 |
| **98779** | 1.320 | 2.32 | 8.84 | -1.143 | 7.52 | 1.44 | 3.09 | 12.72 | -1.50 | 11.28 | 45 | 99 | 0.246 | 0.526 | 0.217 | -1.78 |
| **268260** | 1.017 | 1.74 | 8.72 | -1.036 | 7.70 | 2.21 | 4.44 | 15.33 | -1.49 | 13.12 | 33 | 92 | 0.188 | 0.537 | 0.378 | -1.83 |
| **405839** | 1.184 | 2.12 | 10.51 | -1.155 | 9.33 | 1.61 | 3.09 | 14.21 | -1.34 | 12.61 | 24 | 88 | 0.230 | 0.583 | 0.336 | -1.88 |
| **450840** | 0.885 | 1.74 | 8.79 | -1.225 | 7.91 | 1.74 | 3.86 | 11.94 | -1.59 | 10.20 | 30 | 94 | 0.238 | 0.602 | 0.347 | -1.63 |
| **512612** | 0.747 | 1.55 | 8.65 | -1.253 | 7.90 | 1.81 | 3.86 | 13.57 | -1.54 | 11.76 | 24 | 83 | 0.255 | 0.513 | 0.396 | -1.96 |
| **637368** | 1.131 | 2.12 | 9.94 | -1.220 | 8.81 | 1.21 | 2.51 | 9.94 | -1.40 | 8.73 | 33 | 88 | 0.238 | 0.476 | 0.362 | -1.84 |
| **672200** | 0.991 | 1.74 | 8.26 | -1.073 | 7.27 | 1.39 | 3.28 | 11.85 | -1.63 | 10.46 | 36 | 89 | 0.197 | 0.456 | 0.389 | -1.79 |
| **684814** | 0.925 | 1.93 | 6.93 | -1.332 | 6.01 | 1.65 | 3.48 | 14.09 | -1.50 | 12.44 | 36 | 93 | 0.208 | 0.519 | 0.318 | -1.58 |

TD: Tajima's D. FWH: Fay and Wu's H. fpop1, fpop2: allele frequency of selected site in African and Non-African population, respectively. The first set of statistics (θPi, θW, θH, TD, FWH,$F_{ST}$) summarizes the statistics in a the 4Kb region around the selected site, the second set is in a 8Kb region around the selected site.

**Table X3**. Summary statistics of observed data (first row) and best fitting simulations (rows 2-11) for the CEU data.

| SimNo. | θPi | θW | θH | TD | FWH | θPi2 | θW2 | θH2 | TD2 | FWH2 | fpop1 | fpop2 | $F_{ST}$ | $F_{ST}$ s-sel | $F_{ST}2$ | XPEHH |
|---|---|---|---|---|---|---|---|---|---|---|---|---|---|---|---|---|
| **CEU** | 3.39 | 2.51 | 4.84 | 0.9396 | 1.45 | 5.40 | 4.06 | 11.21 | 0.96289 | 5.814 | 32 | 75 | 0.126 | 0.306 | 0.166 | -1.318 |
| **122975** | 3.13 | 2.70 | 6.38 | 0.4326 | 3.25 | 4.62 | 4.06 | 7.72 | 0.40786 | 3.096 | 23 | 66 | 0.125 | 0.308 | 0.312 | -0.722 |
| **140712** | 2.88 | 1.74 | 5.61 | 1.6344 | 2.73 | 6.31 | 4.06 | 11.47 | 1.61999 | 5.157 | 27 | 64 | 0.141 | 0.235 | 0.140 | -1.447 |

| | | | | | | | | | | | | | | | | |
|---|---|---|---|---|---|---|---|---|---|---|---|---|---|---|---|---|
| **192363** | 2.94 | 4.06 | 4.17 | -0.8020 | 1.23 | 6.43 | 7.15 | 8.20 | -0.31177 | 1.769 | 31 | 83 | 0.160 | 0.427 | 0.187 | -1.414 |
| **379389** | 3.25 | 3.67 | 4.53 | -0.3266 | 1.27 | 5.48 | 5.60 | 6.73 | -0.06739 | 1.248 | 35 | 74 | 0.244 | 0.258 | 0.205 | -1.341 |
| **410678** | 3.01 | 3.48 | 4.62 | -0.3796 | 1.61 | 4.80 | 5.41 | 6.74 | -0.34037 | 1.938 | 21 | 68 | 0.166 | 0.359 | 0.347 | -0.887 |
| **550177** | 3.25 | 2.51 | 6.37 | 0.7948 | 3.11 | 5.68 | 5.41 | 10.89 | 0.14910 | 5.215 | 32 | 81 | 0.104 | 0.386 | 0.133 | -1.300 |
| **560536** | 3.78 | 3.67 | 6.94 | 0.0885 | 3.16 | 5.02 | 5.02 | 7.67 | -0.00074 | 2.646 | 26 | 76 | 0.113 | 0.394 | 0.131 | -1.162 |
| **685594** | 3.82 | 2.90 | 6.10 | 0.8834 | 2.28 | 5.24 | 4.64 | 8.34 | 0.38649 | 3.097 | 29 | 74 | 0.162 | 0.330 | 0.281 | -0.816 |
| **713709** | 3.67 | 3.86 | 5.71 | -0.1471 | 2.04 | 5.21 | 5.21 | 6.14 | -0.00352 | 0.936 | 25 | 69 | 0.191 | 0.319 | 0.155 | -1.379 |
| **751923** | 3.77 | 2.70 | 5.26 | 1.0757 | 1.49 | 5.53 | 5.02 | 9.08 | 0.30031 | 3.555 | 30 | 70 | 0.193 | 0.269 | 0.200 | -1.249 |

TD: Tajima's D. FWH: Fay and Wu's H. fpop1, fpop2: allele frequency of selected site in African and Non-African population, respectively. The first set of statistics (θPi, θW, θH, TD, FWH,$F_{ST}$) summarizes the statistics in a the 4Kb region around the selected site, the second set is in a 8Kb region around the selected site.

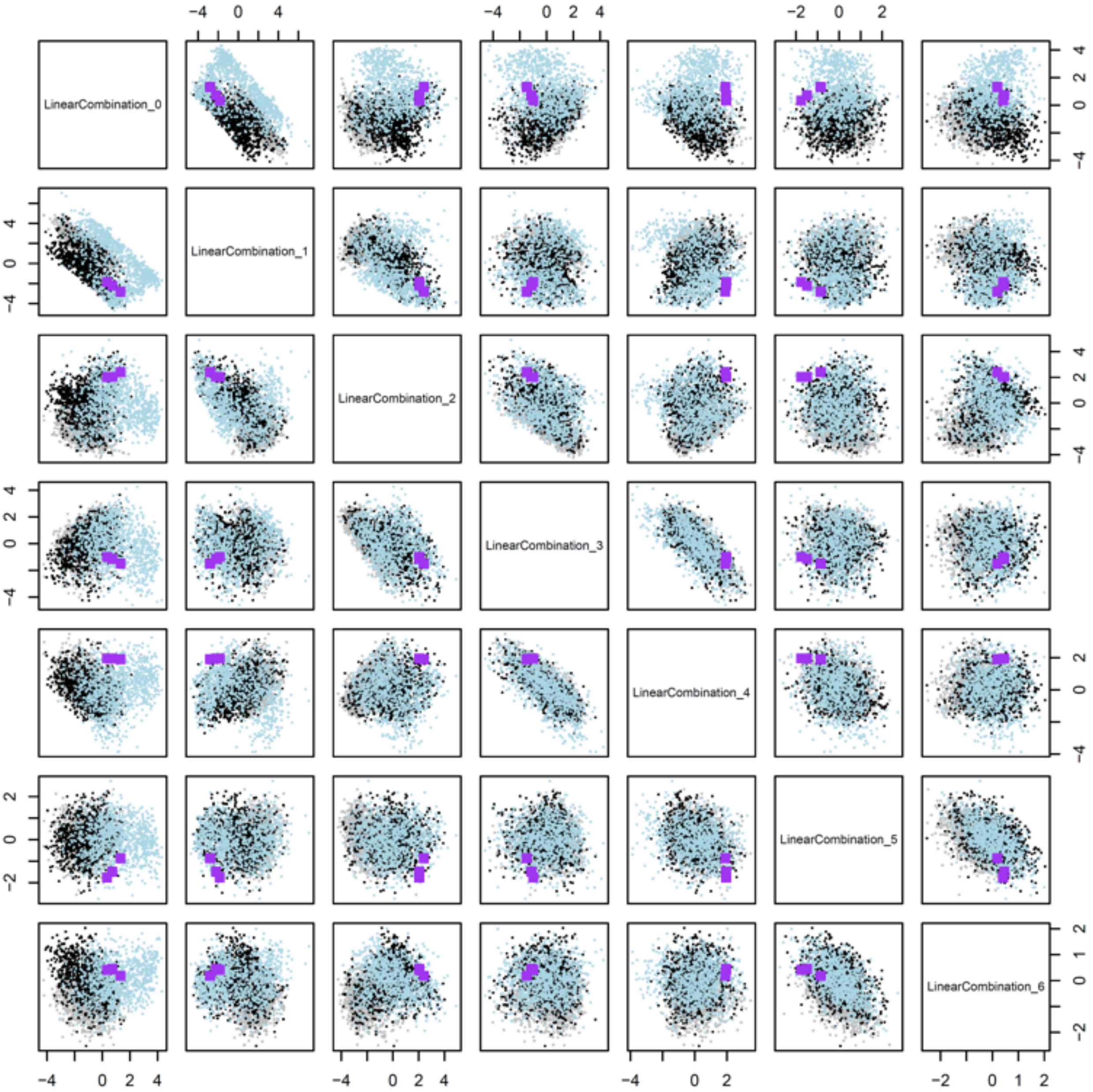


**Figure X3.** Fit of parameter to the Asian populations (CHB, CHS and JPT) to the models using the transformed statistics. The purple dots show the observed statistics from the CHB, CHS and JPT populations. The black, grey and light blue point clouds correspond to 5,000 samples each from the priors for the SSV, NTR and SDN models, respectively.

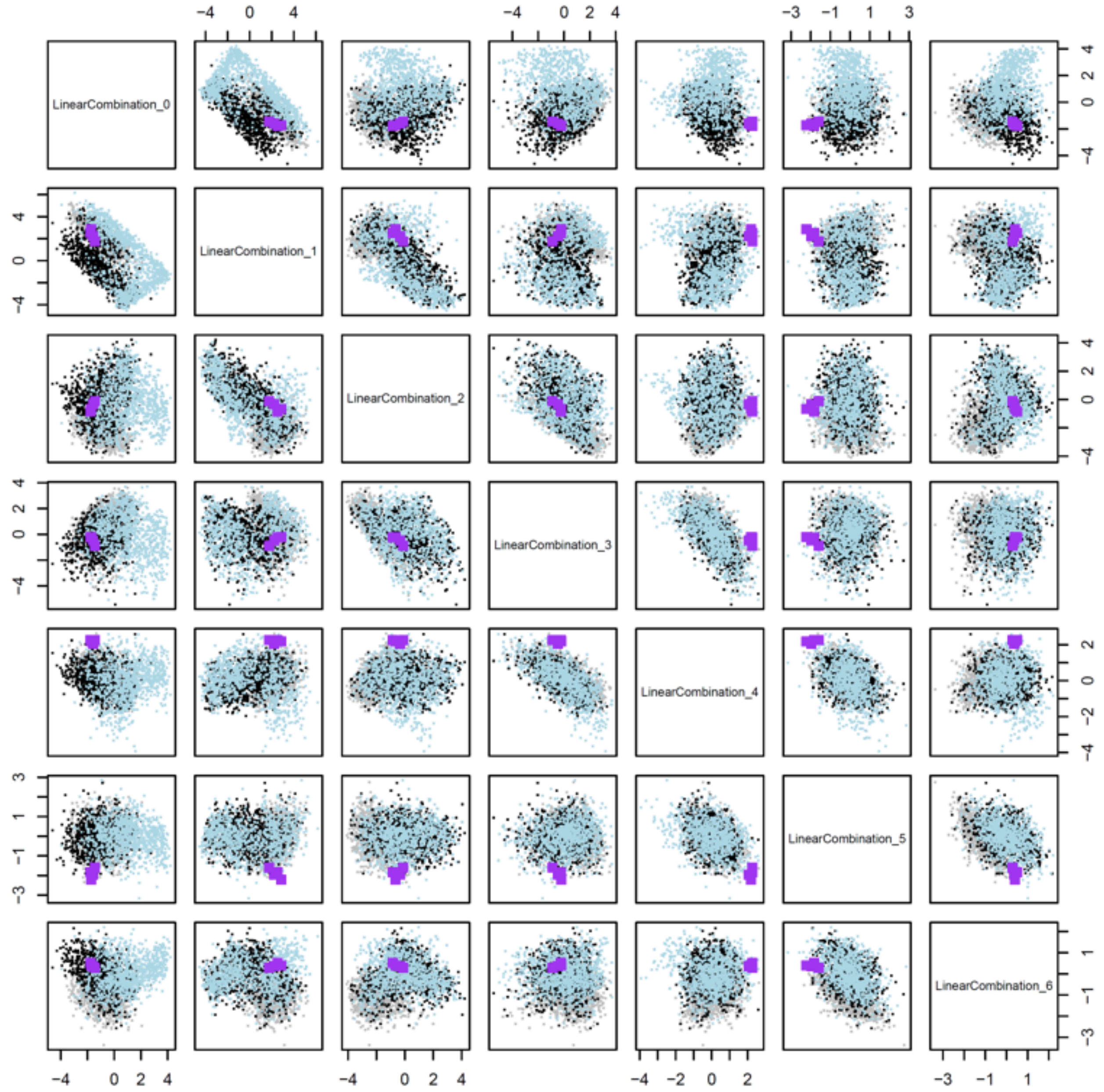


**Figure X4.** Fit of parameter to the European populations (CEU, FIN GBR and TSI) to the models using the transformed statistics. The purple dots show the observed statistics from the CEU, FIN, GBR and TSI populations. The black, grey and light blue point clouds correspond to 5,000 samples each from the priors for the SSV, NTR and SDN models, respectively.

<u>Bias and accuracy of model choice</u>

To infer bias in the model choice, we applied the model choice procedure to 10,000 data sets simulated with parameters drawn randomly from the prior distribution for each of the SDN, SSV and NTR models. We recorded which model the simulations were assigned to in **Figure X5**. We find that we overall have high power to recover the correct model, with 76% of the SSV and 95% of the SDN simulations being assigned correctly under the Asian demographic model, and 70% of the SSV and 97% of the SDN simulations being assigned correctly under the European demographic model. The fact that we have a higher chance of recovering the SDN model compared to the SSV model indicates that the model choice has a slight bias in favor of the SDN

model, which we should keep in mind when interpreting our results. In particular, for the European populations (CEU, FIN, GBR and TSI), our results suggest that the SDN model is favored by a posterior probability of roughly 80%, corresponding to a Bayes factor of around 4 (**Table 3**). According to Jeffrey's interpretation [1], this still provides substantial evidence in favor for the SDN model versus the SSV model. Given the bias, however, we interpret the evidence in Europe more conservatively as non-conclusive. For the Asian populations (CHB, CHS and JPT), the evidence for the SDN model is much stronger, with the posterior probability for the SDN model being between 91.3% and 97.5%, corresponding to a Bayes Factor of at least 10. Thus, even with the bias in mind, these results should be interpreted as substantial evidence for the SDN model.

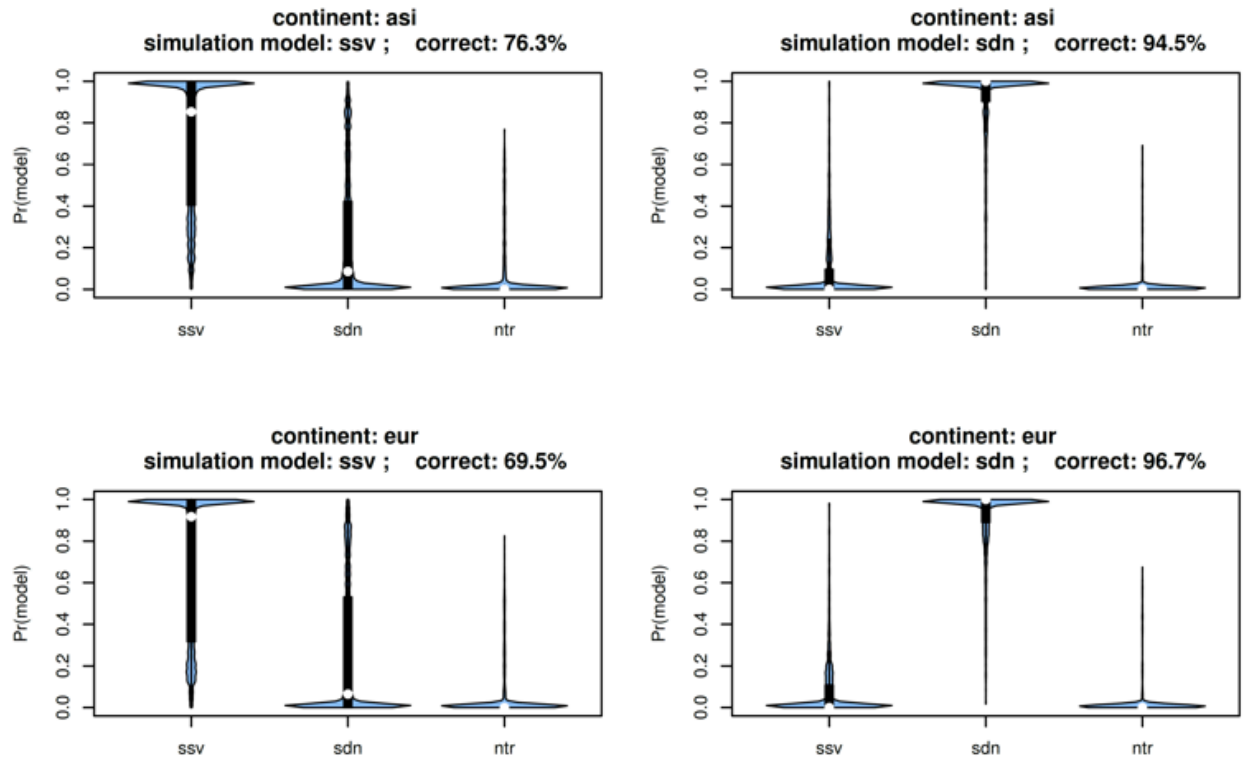


**Figure X5.** Model choice accuracy. We performed the model choice for 10,000 simulated data sets for both the SDN and SSV model using the European (eur) and Asian (asi) demographic model. The violin plots show the distribution of assigned posterior probabilities for each model. The proportion of correctly assigned models is given above the plot.

<u>Bias and accuracy of the parameter estimates</u>

In order to test for a bias in the parameter estimates, we calculated the coverage property [2-4] and relative error of the median. The coverage property is the property that the true parameter value should lay within a confidence interval of the time. Violations may indicate some bias, most likely due to the approximations used in the ABC procedure. In addition, we also report the distributions of error of the median of the distribution, i.e. the distance between the median of our posterior distribution to the parameter the data set was simulated under. This can be used to assess the accuracy of our parameter estimates. The coverage for the three parameters we estimate is given in **Figures X6** and **X7**, and the accuracy in **Figures X8** and **X9**.

We find that both models perform identical, with the coverage and relative error plots being very similar for both demographic models. Coverage deviates slightly from uniform, but not in a magnitude that appears to be worrisome. In the European model, the error distributions are reasonable; the average mean error for the 10,000 simulations is 0.0069 coalescence units for the time the mutation arose. For the selection strength, the mean error is 40.6 and 153 in Africans and Europeans, respectively. Under the Asian model, the errors are 0.0068, 40.9 and 151, which are similar to those in Europeans.

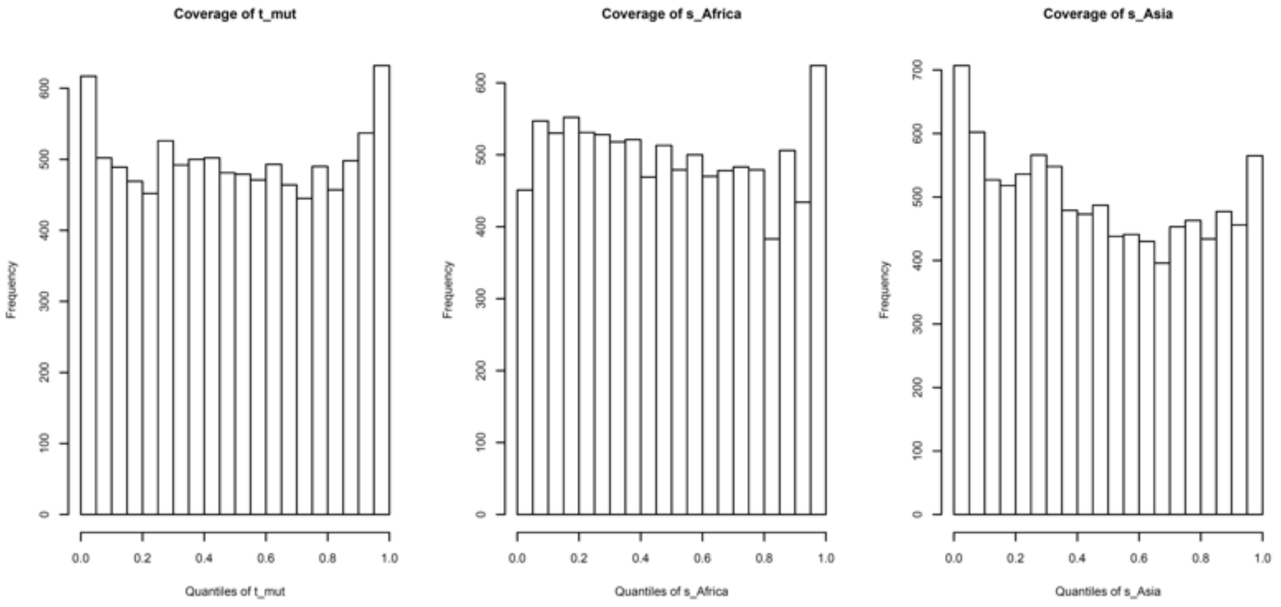


**Figure X6.** Coverage of parameters under the SDN model in the Asian demographic model. If the estimates of the ABC approach are unbiased, the resulting distribution should be uniform. From these plots we infer that the posteriors for $t_{mut}$ (t_mut) are slightly too narrow, $s_A$ (s_Africa) is slightly over- and $s_{NA}$ (s_Asia) slightly underestimated.

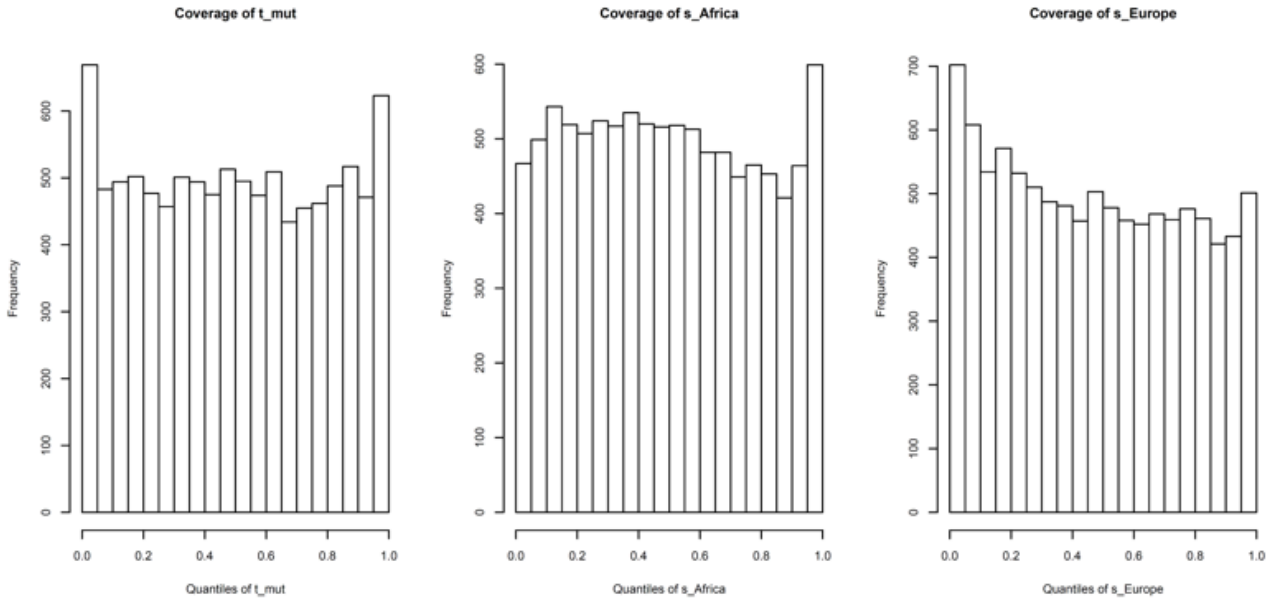


**Figure X7.** Coverage of parameters under the SDN model in the European demographic model. If the estimates of the ABC approach are unbiased, the resulting distribution should be uniform.

From these plots we infer that the posteriors for $t_{mut}$ (t_mut) are slightly too narrow, $s_A$ (s_Africa) is slightly over- and $s_{AN}$ (s_Europe) slightly underestimated.

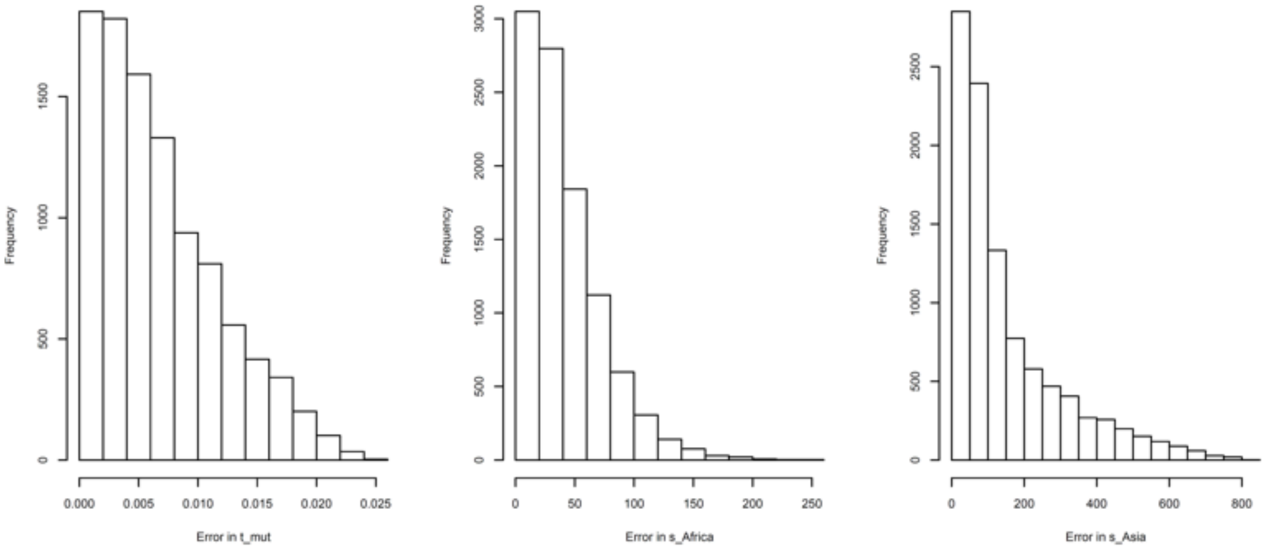


**Figure X8.** Error in parameters under the SDN model in the Asian demographic model. We show the distribution of the relative error of the median for 10,000 simulations.

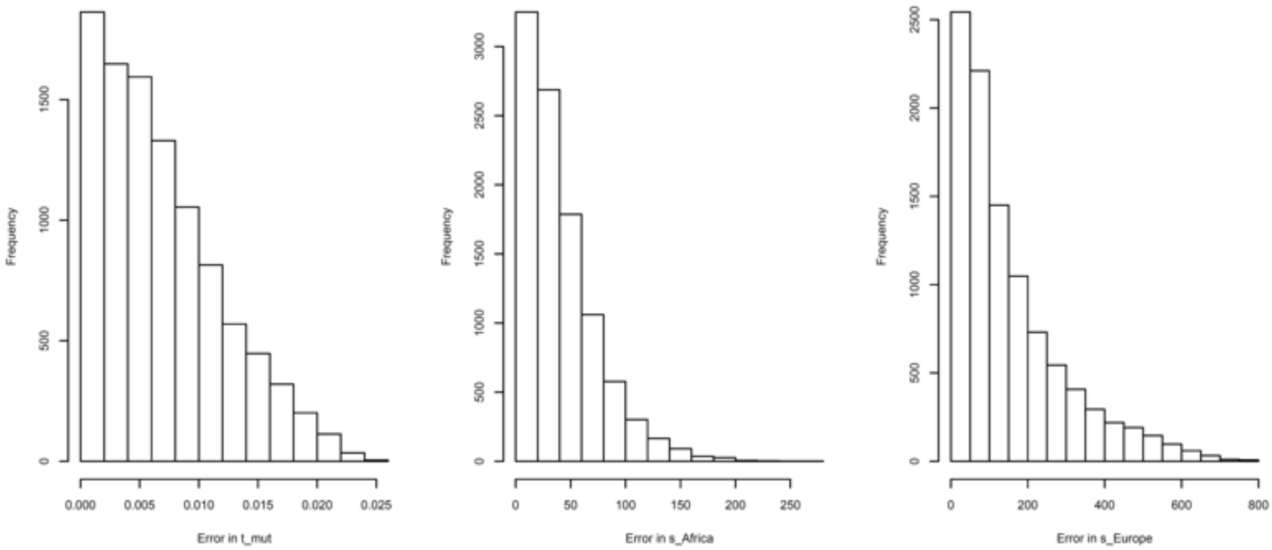


**Figure X9.** Error in parameters under the SDN model in the European demographic model. We show the distribution of the relative error of the median for 10,000 simulations.

**References**

1 Jeffreys H (1998) The theory of probability. Oxford University Press.
2. Cook SR, Gelman A, Rubin DB (2006) Validation of software for Bayesian models using posterior quantiles. Journal of Computational and Graphical Statistics 15.
3. Prangle D, Blum MGB, Popovic G, Sisson SA (2013) Diagnostic tools of approximate Bayesian computation using the coverage property. arXiv preprint arXiv:1301.3166 .
4. Wegmann D, Excoffier L (2010) Bayesian inference of the demographic history of chimpanzees. Mol Biol Evol 27: 1425-1435.
