# Supplementary material for: Selection on a Variant Associated with Improved Viral Clearance Drives Local, Adaptive Pseudogenization of Interferon Lambda 4 (IFNL4)
Source: PLoS Genet. 2014 Oct 16;10(10):e1004681. doi: 10.1371/journal.pgen.1004681 (PMC4199494; doi:10.1371/journal.pgen.1004681)
Supplement: Note S4 — Investigation of the effects of different dominance models in the ABC analysis. (PDF) [file pgen.1004681.s011.pdf]

Supplementary Note 4. Investigation of the effects of different dominance models in the ABC analysis.

As stated in the main text, complete dominance for the derived TT allele is highly unlikely both because of its functional effect and the odds ratios (ORs) of the different genotypes for HCV clearance. The effect of the TT allele might be fully recessive, as both ΔG/ΔG homozygotes and ΔG/TT heterozygotes have functional IFN-λ4, although perhaps in different amounts. A model of additivity is likely both because it is reasonable that the level of IFN-λ4 protein might be relevant, and because heterozygotes have an intermediate phenotype to the two homozygotes with regards to HCV clearance [1,2].

We thus applied our ABC approach to infer the most likely dominance model and to investigate the effects it has on our ABC results. We investigated three reasonable scenarios:

1. Complete recessiveness for TT (h=0).
2. Perfect additivity for ΔG and TT, where heterozygotes have a fitness that is exactly in-between the two homozygotes (h=0.5).
3. Another model that has been proposed based on the OR for HCV clearance given the three genotypes of rs12979860 (T>C) [2], an intronic SNP in strong LD with rs368234815 (**Table 2**) that thus serves as a proxy for the dominance effects of rs368234815. Taking ancestral T/T as baseline, the OR for heterozygotes T/C (OR = 1.48) is closer to the OR of T/T homozygotes than of C/C homozygotes (OR = 3.88), thus the authors propose a supra-additive model of dominance, which is no more than an additive model where h is <0.5. Following these OR values, we estimate an h=0.38 and use it as our supra-additive model.

We performed 500,000 simulations for each of these models (recessive, additive, supra-additive) for both the SDN and the SSV model of selection, and 10,000 simulations under neutrality (NTR). We then run our ABC analysis for model selection, and determined the posterior probability for each of the dominance models regardless of the model of selection (SDN + SSV), and neutrality. In all East Asian populations we observe marginal support for the recessive model (4%), with the support for the additive (56%) and supra-additive (44%) models being very similar (slightly higher for the additive model, **Fig. 5c**). In Europe the results are less clear (as they are less clear in Europeans for all our ABC analyses) but the recessive model still has a lower posterior probability (26%) than both the additive (36%) and supra-additive (38%) models (**Fig. 5c**). Those results are in-line with our expectations as the genetic association with HCV clearance is not consistent with a recessive model.

We then focused on the two additive models and ask whether model selection and parameter estimates are sensitive to the details of these additive models. We analyzed the simulations under the additive and supra-additive models, and performed our ABC analysis. Regarding model selection, in East Asia both additive models provide very similar results, in line with our original ABC analyses. Specifically, in both dominance models SDN is strongly favored in East Asia, with a posterior probability of 97-94% for the additive and 94-90% for the supra-additive model (**Fig. 5b**

and **5d**). In Europe the results are weaker again but consistent, and support the SDN model for the additive (86-74%) and supra-additive (81-67%) models (**Fig. 5b** and **5d**). Thus, based on Bayes factor additive and supra-additive models show strong support for the SDN model in East Asia (Bayes factor ~14), and substantial support for SDN in Europe (Bayes factor ~3). In addition, parameter estimates are also similar in both additive models (additive: **Fig. S5** and supra-additive: **Fig. S6**).

References

1. Prokunina-Olsson L, Muchmore B, Tang W, Pfeiffer RM, Park H, et al (2013) A variant upstream of IFNL3 (IL28B) creating a new interferon gene IFNL4 is associated with impaired clearance of hepatitis C virus. Nat Genet 45: 164.

2. Shebl FM, Pfeiffer RM, Buckett D, Muchmore B, Chen S, et al (2011) IL28B rs12979860 genotype and spontaneous clearance of hepatitis C virus in a multi-ethnic cohort of injection drug users: evidence for a supra-additive association. Journal of Infectious Diseases : jir647.
