# Supplementary material for: Selection on a Variant Associated with Improved Viral Clearance Drives Local, Adaptive Pseudogenization of Interferon Lambda 4 (IFNL4)
Source: PLoS Genet. 2014 Oct 16;10(10):e1004681. doi: 10.1371/journal.pgen.1004681 (PMC4199494; doi:10.1371/journal.pgen.1004681)
Supplement: Note S5 — Investigation of signatures of balancing selection in Africa, and of positive selection for non-synonymous variants on ΔG background. (PDF) [file pgen.1004681.s012.pdf]

Supplementary Note 5. Investigation of signatures of balancing selection in Africa, and of positive selection in non-synonymous variants on ΔG background

Long-term balancing selection maintains several alleles in a population and enhances genetic diversity. That is in contrast with positive or purifying selection, which favor only the best available allele and result in population homogeneity [1]. Given that pseudogenization of *IFNL4* via TT is advantageous in some human populations (at least in East Asia) while, functional *IFNL4* (ΔG) has been evolutionarily maintained throughout the mammal clade, it is possible that both alleles have been under balancing selection in some populations (for example, through temporal or spatial variation in selection). This would fit with the intermediate frequencies observed in African populations (**Figure X10**), if for example an advantageous TT variant carried a locally deleterious phenotype that prevented its rise in frequency in particular populations. Given the function of IFN-λ4 in immune defense, this could be driven by variability in the pathogenic environment [2]. In humans, for example, a scenario where two alleles in the *FLT1* gene fluctuate between being advantageous/deleterious in/out of malaria season has been described [3].

a) *IFNL4* region **b) Control regions**

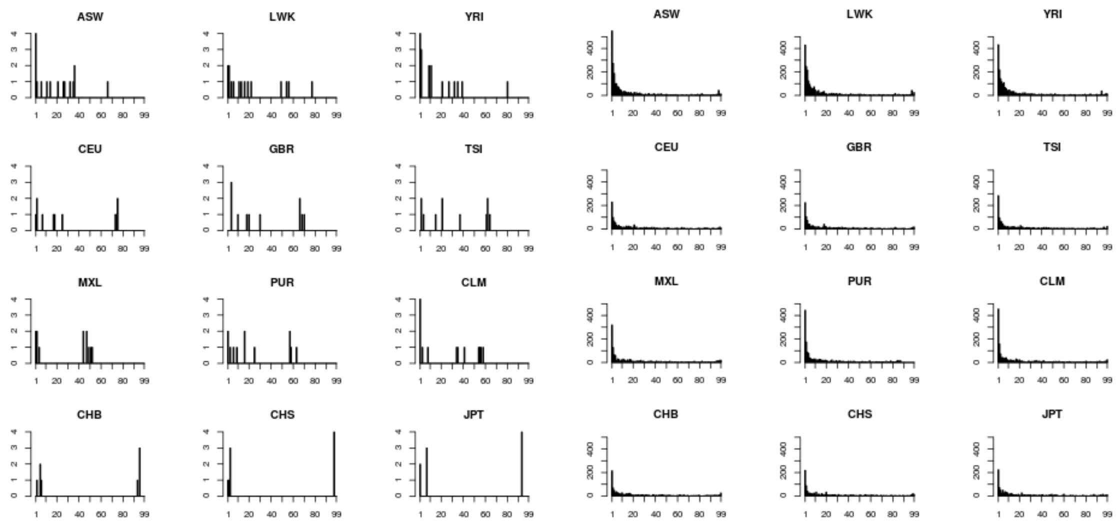

Figure X10. SFS is shown **(a)** for the *IFNL4* region including the 3' and 5' UTRs (chr19:39736954-39739496) and **(b)** for the control regions (pseudogenes, see text below). The SFS is based on 50 individuals for each of the populations but PUR (44 individuals). We show three populations per continent.

To test for additional signatures of natural selection, specifically of balancing selection, in the *IFNL4* region (including the 3' and 5' UTR's; chr19:39736954-39739496) we applied a number of neutrality tests. The HKA [4] test is based on the assumption that under neutrality divergence is proportional to diversity (polymorphism), and both are solely dependent on the mutation rate. Under selection, though, this ratio is affected: long-term balancing selection is expected to result in an enrichment of polymorphisms, while complete (or nearly complete) sweeps of positive selection will result in a deficit of polymorphisms (or an excess or substitutions). To identify such signatures, the HKA test compares the ratio of diversity to divergence of the test-locus and control-loci.

We used pseudogenes as a proxy for neutrality, and we refer to these regions as our 'control regions' (set of pseudogenes described in **Supplementary Note 2**). For these regions and our region of interest diversity was calculated as the number of SNPs, and divergence as the number of substitutions compared to chimpanzee, assessed using the pantro3 lastz alignment from UCSC.

Neutrality tests such as Tajima's D [5] and MWUhigh [6,7] investigate the distribution of allele frequencies (the allele frequency spectrum, SFS, **Figure X10**) of our region of interest compared to neutral expectations. The SFS is affected by natural selection, with balancing selection often resulting in a skew towards intermediate frequency alleles. Both tests are sensitive for such an excess, and thus have power to detect balancing selection. Another SFS-based neutrality test, Fay and Wu [8] (FW), is sensitive to an excess of high-frequency derived SNPs, and thus detects patterns of recent hard sweeps (complete, classical sweeps from a *de novo* mutation).

We estimated significance for all four tests using 10,000 standard neutral coalescent simulations [9]. Because demography affects the SFS and can cause spurious results if not properly accounted for, our simulations are run under a demographic model which includes inferred parameters for populations of African [10], European [10], Asian [10] and American [11] ancestry. In the case of the HKA we simulated demography since the split between chimp and humans using a divergence time of six million years ago. A custom made perl program (Neutrality Test Pipeline) was used to calculate the statistic and its P-value.

Tajima's D and MWUhigh reveal marginal non-significance for European, American, and African populations (**Supplementary Table 7**), which could in principle be interpreted as a signature of either weak balancing selection or balancing selection that maintains alleles at a frequency considerably lower than 50% in the population. Under long-standing balancing selection we also expect an excess of diversity due to the long-term maintenance of two or more alleles in the population; this signature is detected by the HKA test. HKA shows no deviation from neutral expectations (**Supplementary Table 7**), and thus no excess of diversity and no evidence of long-term balancing selection that predates the expected coalescent time under neutrality. Rarely, the excess of intermediate-frequency alleles captured by Tajima's D and MWUhigh can also be the result of an ongoing partial sweep (reviewed in [12]) and by selection from standing variation [13]. So while our results argue against long-term balancing selection in Africa and elsewhere, recent balancing selection or other selective explanations cannot be discarded.

As the transcript with the ΔG allele encodes a functional protein, we assessed whether putatively functional variants in this haplotype show signatures of natural selection. To investigate such a scenario we analyzed the signature of selection for the three described non-synonymous variants in *IFNL4* [14], all residing on the ΔG haplotype. To avoid a confounding impact of the TT haplotype we conditioned on the ΔG haplotype and calculated F_{ST}, XP-EHH and iHS. No signature of natural selection for any of the variants was observed (data not shown). However, all of them are in such low frequencies that these tests have very low power [15,16]. Thus we can not

exclude any beneficial effect for particular variants that could putatively modify the function of the ΔG haplotype.

References

1. Andrés AM (2011) Balancing Selection in the Human Genome Encyclopedia of Life Sciences. John Wiley & Sons, Ltd.
2. Ferrer-Admetlla A, Bosch E, Sikora M, Marquès-Bonet T, Ramírez-Soriano A, et al (2008) Balancing selection is the main force shaping the evolution of innate immunity genes. The Journal of Immunology 181: 1315-1322.
3. Muehlenbachs A, Fried M, Lachowitzer J, Mutabingwa TK, Duffy PE (2008) Natural selection of FLT1 alleles and their association with malaria resistance in utero. Proceedings of the National Academy of Sciences 105: 14488-14491.
4. Hudson RR, Kreitman M, Aguadé M (1987) A test of neutral molecular evolution based on nucleotide data. Genetics 116: 153-159.
5. Tajima F (1989) Statistical method for testing the neutral mutation hypothesis by DNA polymorphism. Genetics 123: 585.
6. Nielsen R, Hubisz MJ, Hellmann I, Torgerson D, Andrés AM, et al (2009) Darwinian and demographic forces affecting human protein coding genes. Genome Res 19: 838-849.
7. Andrés AM, Dennis MY, Kretzschmar WW, Cannons JL, Lee-Lin SQ, et al (2010) Balancing selection maintains a form of ERAP2 that undergoes nonsense-mediated decay and affects antigen presentation. PLoS Genet 6: e1001157.
8. Fay JC, Wu CI (2000) Hitchhiking under positive Darwinian selection. Genetics 155: 1405.
9. Hudson RR (2002) Generating samples under a Wright--Fisher neutral model of genetic variation. Bioinformatics 18: 337-338.
10. Gravel S, Henn BM, Gutenkunst RN, Indap AR, Marth GT, et al (2011) Demographic history and rare allele sharing among human populations. Proceedings of the National Academy of Sciences 108: 11983-11988.
11. Gutenkunst RNAH, Williamson RDA, Bustamante SHA, Carlos C (2009) Inferring the Joint Demographic History of Multiple Populations from Multidimensional SNP Frequency Data. PLoS Genet 5: e1000695.
12. Fu W, Akey JM (2013) Selection and Adaptation in the Human Genome. Annu Rev Genomics Hum Genet .
13. Prezeworski M, Coop G, Wall JD (2005) THE SIGNATURE OF POSITIVE SELECTION ON STANDING GENETIC VARIATION. Evolution 59: 2312-2323.
14. Prokunina-Olsson L, Muchmore B, Tang W, Pfeiffer RM, Park H, et al (2013) A variant upstream of IFNL3 (IL28B) creating a new interferon gene IFNL4 is associated with impaired clearance of hepatitis C virus. Nat Genet 45: 164.
15. Voight BFAK, Wen SA, Pritchard XA, K J (2006) A Map of Recent Positive Selection in the Human Genome. PLoS Biol 4: e72.
16. Sabeti PC, Varilly P, Fry B, Lohmueller J, Hostetter E, et al (2007) Genome-wide detection and characterization of positive selection in human populations. Nature 449: 913-918.
