# Supplementary material for: Selection on a Variant Associated with Improved Viral Clearance Drives Local, Adaptive Pseudogenization of Interferon Lambda 4 (IFNL4)
Source: PLoS Genet. 2014 Oct 16;10(10):e1004681. doi: 10.1371/journal.pgen.1004681 (PMC4199494; doi:10.1371/journal.pgen.1004681)
Supplement: Table S1 — PAML results using either (a) all species or (b) only primates. (PDF) [file pgen.1004681.s013.pdf]

Supplementary Table 1. PAML results using either (**a**) all species and (**b**) only primates.

a)

**Model 1**	**Model 2**	**P-value**	**Estimate model 1**	**Estimate model 2**
One dN/dS	dN/dS < 1	3.45E-27	dN/dS = 0.23	
“	free branch	0.03	“	tree (see Figure 1)
“	primate	0.87	“	dN/dS M2 = 0.24
“	human	0.92	“	dN/dS M2 = 0.23
neutral M1	selection M2	1		
neutral M7	selection M8	0.50		flagged 6W, 103G, 144A
neutral M8a	selection M8	0.81		
neutral M2a	selection M2	1		flagged 136K, 151K

b)

**Model 1**	**Model 2**	**P-value**	**Estimate model 1**	**Estimate model 2**
One dN/dS	dN/dS < 1	2.70E-09	dN/dS = 0.217	
“	free branch	0.08	“	
“	old world monkeys	0.72	“	dN/dS M2 = 0.24
“	human	0.55	“	dN/dS M2 = 0.37
neutral M1	selection M2	0.91		flagged 59Q, 126A, 129G
neutral M7	selection M8	0.69		
neutral M8a	selection M8	0.65		
neutral M2a	selection M2	0.72		flagged 139K, 154K
