# Supplementary material for: Selection on a Variant Associated with Improved Viral Clearance Drives Local, Adaptive Pseudogenization of Interferon Lambda 4 (IFNL4)
Source: PLoS Genet. 2014 Oct 16;10(10):e1004681. doi: 10.1371/journal.pgen.1004681 (PMC4199494; doi:10.1371/journal.pgen.1004681)
Supplement: Table S2 — Frequency of rs368234815 TT allele in the 1000 Genomes dataset and the subset of 50 unrelated individuals per population used for analyses. (PDF) [file pgen.1004681.s014.pdf]

Supplementary Table 2. Frequency of rs368234815 TT allele in the 1000 Genomes dataset and the subset of 50 unrelated individuals per population used for analyses.

Population	Frequency 1000Genomes	Frequency 50 individuals
CHS	0.97	0.97
CHB	0.94	0.95
JPT	0.9	0.93
CEU	0.77	0.75
FIN	0.73	0.71
GBR	0.66	0.68
PUR	0.65	0.65
TSI	0.58	0.61
CLM	0.52	0.55
MXL	0.51	0.47
LWK	0.44	0.49
ASW	0.39	0.36
YRI	0.29	0.32
