# Supplementary material for: Selection on a Variant Associated with Improved Viral Clearance Drives Local, Adaptive Pseudogenization of Interferon Lambda 4 (IFNL4)
Source: PLoS Genet. 2014 Oct 16;10(10):e1004681. doi: 10.1371/journal.pgen.1004681 (PMC4199494; doi:10.1371/journal.pgen.1004681)
Supplement: Table S3 — FST values and corresponding empirical P-values for rs368234815 using different background populations: (a) ASW, (b) LWK (c) GBR, and (d) in the continental comparison. Table (e) shows the empirical P-value of FST for rs368234815 based on genome-wide SNPs with lowest frequency in YRI (compared to ASW and LWK). (PDF) [file pgen.1004681.s015.pdf]

Supplementary Table 3. F_{ST} values and corresponding empirical P-values for rs368234815 using different background populations: **(a)** ASW, **(b)** LWK **(c)** GBR, and **(d)** in the continental comparison. Table **(e)** shows the empirical P-value of F_{ST} for rs368234815 based on genome-wide SNPs with lowest frequency in YRI (compared to ASW and LWK).

a)

**Population vs. ASW**	**F_{ST}**	**F_{ST} P-value**
CHS	0.586	0.002
CHB	0.552	0.003
JPT	0.520	0.004
GBR	0.178	0.056
CEU	0.260	0.025
FIN	0.212	0.042
TSI	0.110	0.115
CLM	0.062	0.164
MXL	0.017	0.434
PUR	0.145	0.043
YRI	-0.006	0.684
LWK	0.026	0.110

b)

**Population vs. LWK**	**F_{ST}**	**F_{ST} P-value**
CHS	0.448	0.016
CHB	0.411	0.021
JPT	0.376	0.026
GBR	0.063	0.253
CEU	0.126	0.139
FIN	0.088	0.203
TSI	0.020	0.498
CLM	-0.002	0.888
MXL	-0.007	0.947
PUR	0.041	0.274
YRI	0.050	0.036
ASW	0.026	0.110

c)

**Population vs. GBR**	**F_{ST}**	**F_{ST} P-value**
CHS	0.448	0.016
CHB	0.411	0.021
JPT	0.376	0.026
GBR	0.063	0.253
CEU	0.126	0.139
FIN	0.088	0.203
TSI	0.020	0.498
CLM	-0.002	0.888
MXL	-0.007	0.947
PUR	0.041	0.274
YRI	0.050	0.036
ASW	0.026	0.110

d)

**Continent vs. Africa**	**F_{ST}**	**F_{ST} P-value**
Asia	0.522	0.005
Europe	0.185	0.054
America	0.048	0.194

e)

**Population vs. YRI low**	**F_{ST}**	**F_{ST} P-value**
CHS	0.6273	0.008
CHB	0.5952	0.01
JPT	0.5637	0.01
GBR	0.22	0.08
CEU	0.3054	0.05
FIN	0.2561	0.08
TSI	0.1471	0.13
CLM	0.0927	0.19
MXL	0.0367	0.36
PUR	0.1854	0.09
LWK	0.0495	0.04
ASW	-0.0059	0.74
