# Supplementary material for: Selection on a Variant Associated with Improved Viral Clearance Drives Local, Adaptive Pseudogenization of Interferon Lambda 4 (IFNL4)
Source: PLoS Genet. 2014 Oct 16;10(10):e1004681. doi: 10.1371/journal.pgen.1004681 (PMC4199494; doi:10.1371/journal.pgen.1004681)
Supplement: Table S4 — Empirical P-values for the XP-EHH analysis for rs368234815 using (a) GBR as background population or (b) in continental comparison. (PDF) [file pgen.1004681.s016.pdf]

Supplementary Table 4. Empirical P-values for the XP-EHH analysis for rs368234815 **(a)** using GBR as background population or **(b)** in continental comparison.

a)

**Population vs. GBR**	**XP-EHH P-value**
CHS	0.04
CHB	0.06
JPT	0.04

b)

**Continent vs. Africa**	**XP-EHH P-value**
Asia	0.004
Europe	0.05
America	0.127
