# Supplementary material for: Selection on a Variant Associated with Improved Viral Clearance Drives Local, Adaptive Pseudogenization of Interferon Lambda 4 (IFNL4)
Source: PLoS Genet. 2014 Oct 16;10(10):e1004681. doi: 10.1371/journal.pgen.1004681 (PMC4199494; doi:10.1371/journal.pgen.1004681)
Supplement: Table S5 — Diversity associated with the TT haplotype in each population, as measured with Watterson's estimator and using only TT/TT homozygous individuals. (PDF) [file pgen.1004681.s017.pdf]

Supplementary Table 5. Diversity associated with the TT haplotype in each population, as measured with Watterson's estimator and using only TT/TT homozygous individuals (nine in each populations, seven in ASW) (see **Supplementary Note 2**).
SNPs: number of SNPs in locus.

	IFNL4 region		Control region	
**Population**	**SNPs**	**Watterson**	**SNPs**	**Watterson**
CHS	4	1.163	988	287.2
CHB	4	1.163	989	287.5
JPT	4	1.163	1021	296.8
GBR	4	1.163	1092	317.5
CEU	4	1.163	1035	300.9
FIN	5	1.454	1081	314.3
TSI	5	1.454	1066	309.9
CLM	4	1.163	1131	328.8
MXL	4	1.163	1107	321.8
PUR	4	1.163	1217	353.8
ASW	5	1.572	1310	411.9
LWK	11	3.198	1525	443.4
YRI	8	2.326	1482	430.9
