# Supplementary material for: Selection on a Variant Associated with Improved Viral Clearance Drives Local, Adaptive Pseudogenization of Interferon Lambda 4 (IFNL4)
Source: PLoS Genet. 2014 Oct 16;10(10):e1004681. doi: 10.1371/journal.pgen.1004681 (PMC4199494; doi:10.1371/journal.pgen.1004681)
Supplement: Table S6 — ABC results and inferred parameter estimates for (a) the SSV model and (b) the neutral model. (PDF) [file pgen.1004681.s018.pdf]

Supplementary Table 6. ABC results and inferred parameter estimates for the (**a**) SSV model and (**b**) the neutral model.
P: posterior probability, t_{mut}: time of selection (based on a generation time of 25yr), f_0: frequency of site at selection initiation, S_{NA}: selection coefficient in the non-African population. The 95% confidence interval (CI) is shown in brackets.

a) SSV

Pop	P (in %)	t_{mut} (in ky)	t_{mut} (in 4Ne)	f_0	S_{NA} (in %)	S_{NA} (in 2Ne*s)
CHS	2.5	42.0 (25.6-50.4)	0.042 (0.026-0.050)	0.148 (0.050-0.197)	1.17 (0.18-4.12)	235 (36-824)
CHB	4.9	41.1 (24.3-50.4)	0.041 (0.024-0.050)	0.159 (0.074-0.198)	1.08 (0.16-3.89)	217 (31-779)
JPT	8.7	41.0 (24.0-50.3)	0.041 (0.024-0.050)	0.162 (0.084-0.198)	1.11 (0.16-3.97)	222 (31-794)
CEU	14.7	39.6 (22.2-50.3)	0.040 (0.022-0.050)	0.154 (0.065-0.197)	1.07 (0.11-3.49)	215 (21-699)
GBR	15.2	40.8 (22.6-50.4)	0.041 (0.023-0.050)	0.151 (0.059-0.197)	0.79 (0.03-2.84)	159 (6-568)
TSI	18.1	40.6 (22.5-50.4)	0.041 (0.022-0.050)	0.152 (0.060-0.197)	0.74 (0.03-2.64)	147 (6-528)
FIN	26.0	39.9 (22.3-50.3)	0.040 (0.022-0.050)	0.151 (0.059-0.197)	0.98 (0.06-3.32)	197 (11-664)

b) NTR

Population	P (in %)
CHS	8.36E-10
CHB	3.30E-07
JPT	4.08E-06
CEU	0.025
GBR	0.004
TSI	0.007
FIN	0.043
