# Supplementary material for: Selection on a Variant Associated with Improved Viral Clearance Drives Local, Adaptive Pseudogenization of Interferon Lambda 4 (IFNL4)
Source: PLoS Genet. 2014 Oct 16;10(10):e1004681. doi: 10.1371/journal.pgen.1004681 (PMC4199494; doi:10.1371/journal.pgen.1004681)
Supplement: Table S7 — Tajima's D (TD), HKA, and MWUhigh results. (PDF) [file pgen.1004681.s019.pdf]

**Supplementary Table 7.** Tajima's D (TD), HKA, and MWUhigh results. All tests were performed for three populations per continent.

| Population | TD | TD P-value | HKA P-value | MWUhigh P-value |
|---|---|---|---|---|
| CHS | -1.82 | 0.98 | 0.95 | 0.90 |
| CHB | -1.23 | 0.86 | 0.81 | 0.71 |
| JPT | -1.12 | 0.82 | 0.90 | 0.73 |
| GBR | 1.36 | 0.08 | 0.92 | 0.06 |
| CEU | 0.75 | 0.19 | 0.93 | 0.19 |
| TSI | 1.63 | 0.05 | 0.99 | 0.04 |
| CLM | 1.26 | 0.07 | 0.94 | 0.10 |
| MXL | 1.60 | 0.04 | 0.95 | 0.03 |
| PUR | 0.97 | 0.11 | 0.78 | 0.08 |
| ASW | 0.90 | 0.04 | 0.68 | 0.07 |
| LWK | 0.60 | 0.08 | 0.66 | 0.06 |
| YRI | 0.07 | 0.20 | 0.93 | 0.23 |
