# Supplementary material for: Selection on a Variant Associated with Improved Viral Clearance Drives Local, Adaptive Pseudogenization of Interferon Lambda 4 (IFNL4)
Source: PLoS Genet. 2014 Oct 16;10(10):e1004681. doi: 10.1371/journal.pgen.1004681 (PMC4199494; doi:10.1371/journal.pgen.1004681)
Supplement: Table S8 — IFNL4 orthologous exons retrieved through BLAT search with human reference IFNL4-ΔG (for primate species) and the panda ortholog (for non-primate species). (PDF) [file pgen.1004681.s020.pdf]

Supplementary Table 8. *IFNL4* orthologous exons retrieved through BLAT search with human reference *IFNL4*-ΔG (for primate species) and the panda ortholog (for non-primate species). Note, other species (Gorilla, Gibbon, Rabbit, Squirrel, Cow) did not show a functional ORF.

**Species**	**Genome**	**Locus**
Chimpanzee	CGSC 2.1.3/panTro3	chr19:44470368-44471812
Orangutan	WUGSC 2.0.2/ponAbe2	chr19:40176375-40177819
Rhesus macaque	MGSC Merged 1.0/rheMac2	chr19:45682211-45683649
Squirrel monkey	Broad/saiBol1	JH378281:400232-401677
Marmoset	WUGSC 3.2/calJac3	chr22:32548950-32550394
Dog	Broad/canFam2	chr1:113939036-113940582
Panda	BGI-Shenzhen 1.0/ailMel1	GL193591.1:278303-279771
Pig	SGSC Sscrofa10.2/susScr3	chr14:143802216-143803657
Megabat	Broad/pteVam1	scaffold_8982:28501-29915
Elephant ortholog 1	Broad/loxAfr3	scaffold_99:2541944-2543345
Elephant ortholog 2	Broad/loxAfr3	scaffold_99:2549984-2551385
Mouse	GRCm38/mm10	absent
Rat	Baylor 3.4/rn4	absent
