# Supplementary material for: Correction: Selection on a Variant Associated with Improved Viral Clearance Drives Local, Adaptive Pseudogenization of Interferon Lambda 4 (IFNL4)
Source: PLoS Genet. 2014 Nov 19;10(11):e1004882. doi: 10.1371/journal.pgen.1004882 (PMC4237317; doi:10.1371/journal.pgen.1004882)
Supplement: File S1 — Originally published, uncorrected article. (PDF) [file pgen.1004882.s001.pdf]

OPEN ACCESS Freely available online

PLOS | GENETICS

Selection on a Variant Associated with Improved Viral Clearance Drives Local, Adaptive Pseudogenization of Interferon Lambda 4 (*IFNL4*)

Felix M. Key[1], Benjamin Peter[2], Megan Y. Dennis[3], Emilia Huerta-Sánchez[4], Wei Tang[5], Ludmila Prokunina-Olsson[5], Rasmus Nielsen[2], Aida M. Andrés[1]*

1 Department of Evolutionary Genetics, Max Planck Institute for Evolutionary Anthropology, Leipzig, Germany, **2** Department of Integrative Biology, University of California Berkeley, Berkeley, California, United States of America, **3** Department of Genome Sciences, University of Washington School of Medicine, Seattle, Washington, United States of America, **4** School of Natural Sciences, University of California Merced, Merced, California, United States of America, **5** Laboratory of Translational Genomics, Division of Cancer Epidemiology and Genetics, National Cancer Institute, National Institutes of Health, Bethesda, Maryland, United States of America

Abstract

Interferon lambda 4 gene (*IFNL4*) encodes IFN-λ4, a new member of the IFN-λ family with antiviral activity. In humans *IFNL4* open reading frame is truncated by a polymorphic frame-shift insertion that eliminates IFN-λ4 and turns *IFNL4* into a polymorphic pseudogene. Functional IFN-λ4 has antiviral activity but the elimination of IFN-λ4 through pseudogenization is strongly associated with improved clearance of hepatitis C virus (HCV) infection. We show that functional IFN-λ4 is conserved and evolutionarily constrained in mammals and thus functionally relevant. However, the pseudogene has reached moderately high frequency in Africa, America, and Europe, and near fixation in East Asia. In fact, the pseudogenizing variant is among the 0.8% most differentiated SNPs between Africa and East Asia genome-wide. Its raise in frequency is associated with additional evidence of positive selection, which is strongest in East Asia, where this variant falls in the 0.5% tail of SNPs with strongest signatures of recent positive selection genome-wide. Using a new Approximate Bayesian Computation (ABC) approach we infer that the pseudogenizing allele appeared just before the out-of-Africa migration and was immediately targeted by moderate positive selection; selection subsequently strengthened in European and Asian populations resulting in the high frequency observed today. This provides evidence for a changing adaptive process that, by favoring IFN-λ4 inactivation, has shaped present-day phenotypic diversity and susceptibility to disease.

Citation: Key FM, Peter B, Dennis MY, Huerta-Sánchez E, Tang W, et al. (2014) Selection on a Variant Associated with Improved Viral Clearance Drives Local, Adaptive Pseudogenization of Interferon Lambda 4 (*IFNL4*). PLoS Genet 10(10): e1004681. doi:10.1371/journal.pgen.1004681

Editor: Jonathan K. Pritchard, Stanford University, United States of America

Received April 9, 2014; **Accepted** August 18, 2014; **Published** October 16, 2014

This is an open-access article, free of all copyright, and may be freely reproduced, distributed, transmitted, modified, built upon, or otherwise used by anyone for any lawful purpose. The work is made available under the Creative Commons CC0 public domain dedication.

Data Availability: The authors confirm that all data underlying the findings are fully available without restriction. All data is available from 1000 Genomes, ftp://ftp.1000genomes.ebi.ac.uk/vol1/ftp/phase1/; GENCODE, http://pseudogene.org/psidr/; HapMap, http://hapmap.ncbi.nlm.nih.gov.

Funding: FMK and AMA are funded by the Max Planck Society. MYD is supported by the National Institute of Neurological Disorder and Stroke of the U.S. National Institutes of Health (award K99NS083627). WT and LPO are supported by the Intramural Research Program of the NCI/NIH. RN and EHS are supported by research grants R01HG003229 (RN) and R01HG003229-08S2 (EHS) from the U.S. NIH. The funders had no role in study design, data collection and analysis, decision to publish, or preparation of the manuscript.

Competing Interests: The authors have declared that no competing interests exist.

* Email: aida_andres@eva.mpg.de

Introduction

Interferon-lambda (IFN-λ) proteins induce antiviral effectors in host target cells and have a crucial role in immune defense against pathogens [1]. The *IFNL* family classically included three genes (*IFNL1*, *IFNL2*, and *IFNL3*; formerly *IL29*, *IL28A*, *IL28B*, respectively) located within a 50 kb region of chromosome 19 [2,3]. Several intergenic variants within the *IFNL* cluster had been identified as showing remarkable association with clearance of hepatitis C virus (HCV) [4–6], which is worldwide responsible for ~170 million infections and over 350,000 deaths per year [7,8]. The underlying functional basis of this association remained unclear despite numerous efforts to identify functional consequences of these variants [9–16].

An additional member of the IFN-λ family has recently been discovered: IFN-λ4, which bears only 30% amino acid identity with the other IFN-λs and is encoded by the *IFNL4* gene, also located within the *IFNL* locus [2,17]. IFN-λ4 shows similar antiviral activity like IFN-λ3 but as it shows limited secretion it might also act intracellularly, unlike the other IFN-λs [18]. A compound di-nucleotide exonic variant (rs368234815, ΔG>TT) in *IFNL4* causes a frame-shift of its open reading frame and results in the polymorphic pseudogenization of *IFNL4* - the polymorphic loss of IFN-λ4 protein [17]. The existence of *IFNL4* was not even computationally predicted because the human reference genome contains the TT allele and lacks the *IFNL4* open reading frame [17]. Remarkably, the derived TT allele not only eliminates IFN-λ4, but it also shows the strongest genetic association reported to date with improved spontaneous and treatment-induced HCV clearance [17,19,20].

The function of IFN-λ proteins is crucial for response to pathogens and this locus has evolved under natural selection, with signatures of positive selection being described in the three classical *IFNL* genes (*IFNL1-3*) [21]. However, that analysis did not cover the *IFNL4* gene, nor the frame-shift rs368234815 variant, which were then unknown [21]. Therefore, the

Author Summary

The genetic association with clearance of Hepatitis C virus (HCV) is one of the strongest and most elusive known associations with disease. The genetic variant more strongly associated with improved HCV clearance inactivates the recently discovered *IFNL4* gene, which encodes for antiviral IFN-λ4 protein, and turns it into a polymorphic pseudogene. We show that functional IFN-λ4 is conserved and functionally important in mammals. In humans though the inactivating mutation appeared in Africa just before the out-of-Africa migration and quickly became advantageous, with the strength of selection (the degree of advantage) varying across human groups. In particular, selection became stronger out of Africa and was strongest in East Asia, raising the frequency of the pseudogene and resulting in the virtual loss of functional IFN-λ4 protein in several Asian populations. Although the environmental force driving selection is unknown, this process resulted in variable clearance of HCV in modern human populations. The complex selective history of *IFNL4*-inactivating allele has thus shaped present-day heterogeneity across populations not only in genetic variation, but also in relevant phenotypes and susceptibility to disease.

evolutionary history of this interesting functional variant and its influence on the local signatures of selection remained unknown.

Here we report an in-depth comparative and population genetic analysis that focuses on *IFNL4* and the rs368234815 polymorphism. We show that the functional IFN-λ4 protein is under purifying selection in mammals, while in humans the *IFNL4* pseudogenizing TT allele carries strong signatures of positive selection. We use a new Approximate Bayesian Computation (ABC) approach [22,23] to provide evidence of a complex selective history of the TT allele, which involves changes in selective strength across human populations. This selective process had important implications in present-day phenotypic diversity and susceptibility to disease.

Results

Functional IFN-λ4 is strongly conserved in mammals

The *IFNL4* gene is present in most mammals analyzed, although it is absent in mouse and rat (Methods). To understand the evolutionary conservation of *IFNL4* we performed a comparative analysis of the *IFNL4* coding sequences from a representative set of mammals (N = 12). The overall dN/dS (non-synonymous to synonymous substitution ratio) is 0.23 across mammals and 0.22 across primates (Figure 1), indicative of purifying selection maintaining the sequence and function of the protein. Notably, all individual branches except squirrel monkey have dN/dS<1 and no model of protein evolution supported dN/dS>1 in specific branches or sites (Table S1). This reveals strong evolutionary conservation of IFN-λ4 in mammals, reflecting its functional relevance.

Strong population differentiation for the TT allele

The selective constraint on IFN-λ4 contrasts with the pseudogenization of the gene in humans through the derived TT allele [17]. The multiple-species alignment shows that ΔG is the conserved, ancestral allele and TT is the derived human-specific allele. The mutational process from ΔG to TT in humans is unclear, but only these two forms have been observed, so they should be considered as two alleles of a di-nucleotide variant (Methods). The TT allele shows considerable frequency variation across human groups. The 1000 Genomes data [24] reveals a gradient in frequency that rises from Africa (0.29–0.44) to Europe (0.58–0.77) and the New World (0.51–0.65), and reaches near fixation in East Asia (0.94–0.97) (Figure 2, Table S2, full population names in Methods).

Population differentiation can be quantified with the fixation index F_{ST} [25], a measure of the pairwise level of differentiation in allele frequencies. We used Yoruba (YRI) as the background population because it has the lowest frequency of the derived TT allele in Africa. To put these values in the context of genome-wide population differences, F_{ST} was also calculated for every SNP in the 1000 Genomes dataset. For the TT allele the largest F_{ST}, 0.63, corresponds to Southern Han Chinese (CHS) versus YRI, which places the TT allele in the 0.5% tail of the empirical genomic distribution of CHS-YRI F_{ST} (Fig. 3A, Table 1). F_{ST} is also in the 0.8% tail of the genomic distribution for the other East Asian populations (CHB, JPT, Fig. 3B and C, Table 1), and in the 4% tail for Europeans (CEU) and one African population, Luhya (LWK) (Table 1, Fig. S1). These results remain significant when other populations were used as background and in continental comparisons, and when the genome-wide distribution was restricted to SNPs with the lowest frequency in Yoruba (Table S3). Therefore, rs368234815 is among the 0.8% most differentiated SNPs between African and East Asians, and among the 12% most differentiated SNPs between African and European populations.

The TT allele resides in a population-specific extended haplotype

The unusually high population differentiation of the TT allele is compatible with a scenario of recent population-specific natural selection. Under certain selection models such high differentiation should be accompanied by extended haplotype homozygosity in the populations experiencing selection, but not in other populations. We evaluated such a signature with the cross population extended haplotype homozygosity test [26] (XP-EHH), which was calculated across the genome relative to the Yoruba population. The XP-EHH value for the TT allele is in the 0.5% tail of the empirical distribution for East Asian populations ($p = 0.003$, 0.005, and 0.003, for CHS, CHB, and JPT, Fig. 3A–C, Table 1), and the signal remains significant when calculated relative to a European population (GBR) and in analyses at the continental level (Table S4). In addition, some non-Asian populations show marginally significant signatures of positive selection too (CEU, PUR, LWK, Table 1, Fig. S1). Similar results were obtained with iHS, a statistic that explores haplotype homozygosity within a single population [27] (Table 1) (although iHS lacks power when population frequency is very high, like in Asia). The unusual allele-specific haplotype homozygosity is evident in Figure 3D, which shows the haplotype structure of the locus in one African, one European, and one East Asian population (for all populations see Fig. S3). We note that F_{ST} shows very weak correlation with both XP-EHH and iHS in the genome ($r = 0.12$ and $r = -0.08$ Spearman rank test, respectively, although the large number of data points makes these weak correlations significant, P-value< 2.2e-16). Therefore, the F_{ST} and XP-EHH/iHS observations can be considered largely independent.

Finally, not only rs368234815 itself but also its genetic locus shows signatures of recent positive selection, with significant Fay and Wu's H test [28] (FW), which detects an excess of high-frequency derived alleles in the region (Table 1). Together, the combined signatures of F_{ST}, XP-EHH, iHS and FW provide strong evidence for the action of natural selection rapidly

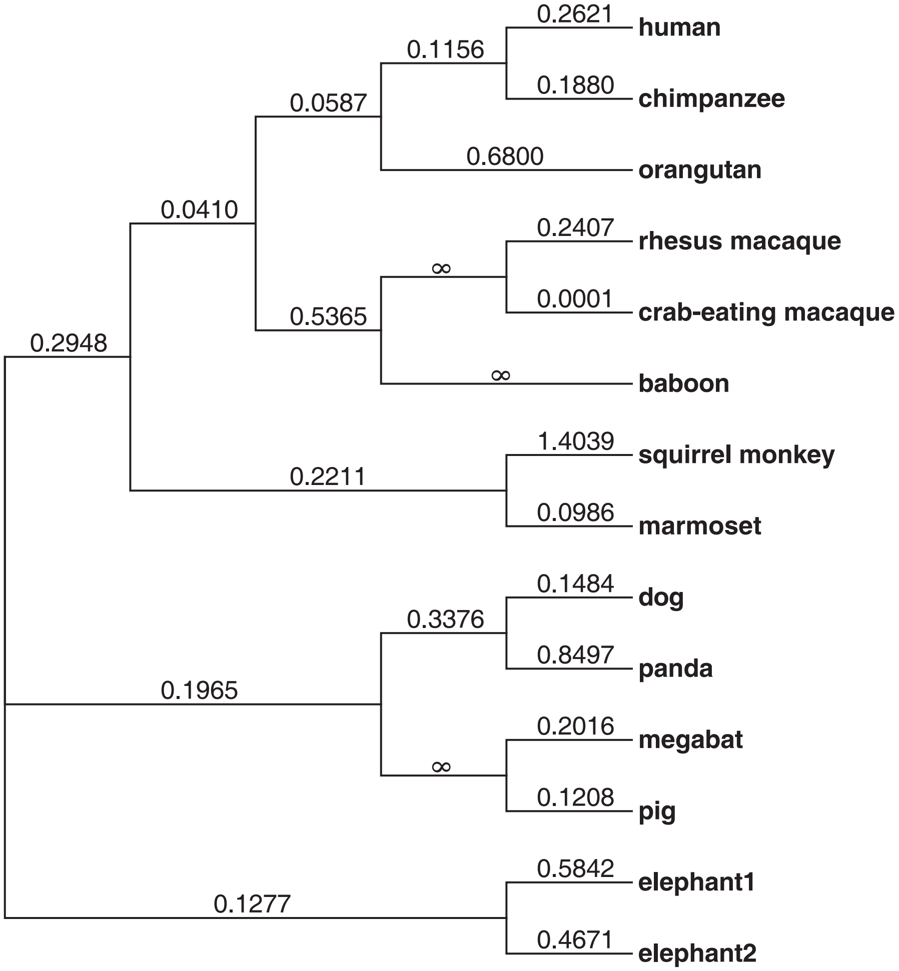

Figure 1. Phylogenetic tree showing the dN/dS ratio of each lineage analyzed.
doi:10.1371/journal.pgen.1004681.g001

increasing the frequency of the TT allele in East Asia. The signature outside Asia is less clear, with most populations showing significant signatures of selection for a subset of the tests performed.

Cumulative evidence that TT allele drives the signatures of selection

A classical problem in population genetics is the identification of the genetic variant responsible for a selection signal. High linkage disequilibrium (LD) in the region surrounding *IFNL4* (Table 2, Fig. 3D, Fig. 4, Fig. S7) hampers the distinction of signatures across all the linked variants, making it difficult to identify the causal variant. We conclude that rs368234815 is the most likely variant driving the signatures of selection, based on three lines of evidence: (1) its functionality and phenotypic consequences, (2) its genetic association with viral clearance, which reflects its effect on fitness, and (3) its signatures of selection.

First, the TT allele has a clear phenotypic consequence as it leads to abrogation of IFN-λ4. This is in contrast with other variants in the locus for which no conclusive functional data has been reported despite numerous efforts [9–14]. Second, of all variants in the *IFNL* region, rs368234815 shows the strongest genetic association with spontaneous and treatment-induced HCV clearance in African Americans [17,19]; in Europeans and Asians the strong LD across the region results in comparable associations for many variants [15–17,20,29] (Table 2, Fig. S2, Fig. S7). Third, of all protein-coding or HCV-associated variants in this locus, rs368234815 shows the strongest combined signatures of positive selection in East Asians (Fig. 3A–C, Fig. S1 and S2, Table 2). Only one other polymorphism (intergenic rs8109886, located upstream of *IFNL4*, Fig. 4), shows signals of selection comparable to rs368234815 (Fig. S2, Table 2). No function has been ascribed to this variant despite a moderate HCV association that is likely due to linkage to TT [6,17,30] (Table 2 and Fig. 4), making it *a priori* a less likely candidate for selection. Indeed, simulations of the evolutionary process showed that the large frequency change of rs8109886 can be explained by linkage to the TT allele alone (Note S1).

We also put rs368234815 in the context of the signatures of selection in the larger genomic region. *IFNL4* is located upstream of *IFNL3* in a region of moderate LD that is separated from the *IFNL1/IFNL2* locus by a recombination hotspot (Fig. S7, Table 2). Manry et al. [21] identified signatures of recent positive selection in all three original *IFNL* genes (*IFNL1-3*) but neither *IFNL4* nor the rs368234815 variant were known at that time and thus they were not considered. The recombination hotspot breaks LD between the *IFNL1/INFL2* locus and the *IFNL3/IFNL4* locus (Fig. S7, Table 2), showing that these signatures are in all likelihood independent, as suggested by Manry et al. [21]. There is moderate LD between *IFNL4* and *IFNL3*, with an average r^2

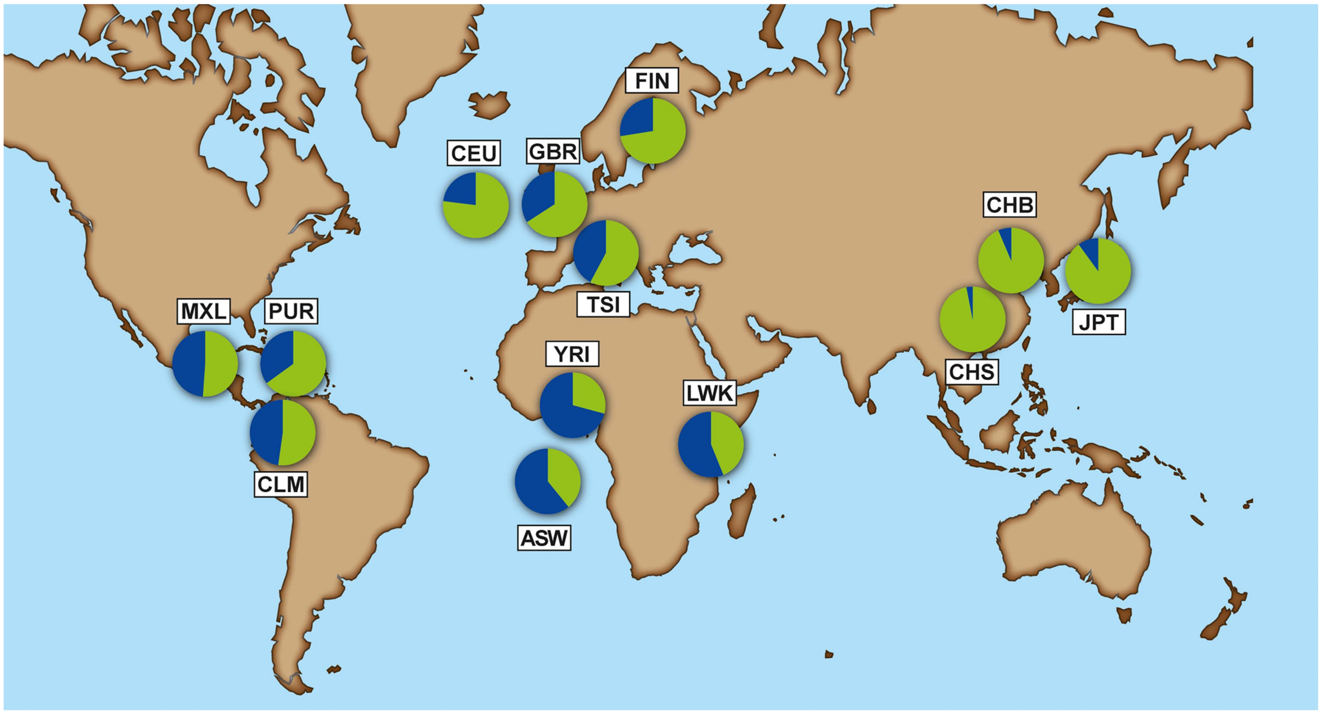

Figure 2. Allele frequency of rs368234815 - ΔG allele (blue) and TT allele (green) for each population from the 1000 Genomes dataset. American populations of European and African origin (CEU, ASW) are placed near the geographic area of origin. For full population names see Methods.
doi:10.1371/journal.pgen.1004681.g002

between rs368234815 and *IFNL3* SNPs of 0.18 in CEU and of 0.44 in CHS (see also Fig. S7). So, the selection signatures in *IFNL3* and *IFNL4* may not be independent. In fact, the seven SNPs identified by Manry et al. [21] (detailed in Table 2 and Fig. 4) have (1) weaker signatures of selection; (2) unclear functional effects, and (3) weaker association with HCV clearance than the TT allele in Africa (Table 2, Figure 4). Also, those that show some signatures of selection have high to moderate LD with rs368234815 (Table 2), with LD broken mostly by a few recombination events in the ancestral haplotype (Note S1). Taken together, these lines of evidence confirm that *IFNL1/2* and *IFNL3/IFNL4* have likely been independently targeted by positive selection in recent human history, as suggested by Manry et *al.* [21], and highlight rs368234815 TT as the most likely selected allele in its region.

Mode and *tempo* of positive selection on the TT allele

The classical model of positive selection involves selection on a *de novo* mutation (SDN), a so-called hard sweep, where a new mutation immediately becomes beneficial and selected (reviewed in [31]). This scenario is difficult to reconcile with our observations, because unequivocal signatures of selection are observed only in East Asians but the TT allele is common worldwide. The TT-carrying haplotype harbors the highest genetic diversity in Africa indicating that it arose there before the out-of-Africa dispersion (Note S2, Table S5), a result that is consistent with the *IFNL4* haplotype network (Fig. S4). Under SDN, only a model where selection begins weak in Africa and becomes stronger outside of Africa could explain our observations (Fig. 5A). An alternative model is selection from standing variation (SSV), also known as a soft sweep (reviewed in [31]). In this scenario an existing neutral or nearly neutral allele becomes advantageous, for example upon environmental change (Fig. 5A).

To disentangle the most likely model of selection for the TT allele we applied a modified version of a recently published ABC approach [23], which we extended to be able to analyze two-population models. In brief, we match millions of simulations under the different models to a summary of the observed genetic data in the *IFNL4* region, and use the best matching simulations for further inferences. Under reasonable assumptions we expect the most realistic selection model to produce the closest simulations to real data, and thus simulations can be used to make inferences about the selective history of the allele [23] including the model of selection and relevant parameters (Note S3). While the method relies on some assumptions (e.g. correct demographic and dominance models) this approach has been shown to be robust and to have high power to recover the correct selection scenario [23]. We assess that we overall have high power to recover the correct model, with 76% of the SSV and 95% of the SDN simulations being assigned correctly under the East Asian demographic model, and 70% of the SSV and 97% of the SDN simulations being assigned correctly under the European demographic model (Note S3). The slight bias observed was considered when interpreting the results. For our analysis we consider three models: neutrality (no selection), selection from a *de novo* mutation (SDN) and selection from standing variation (SSV) (Fig. 5A).

In East Asian populations we obtain negligible support for neutrality and very strong support for the SDN model (Fig. 5B, Table 3, Table S6). Results in Europeans are also consistent with the SDN model, although the weaker signals of selection and the slight bias observed above make these results less conclusive (Table 3, Note S3, Fig. S5, Table S6). The posterior probability

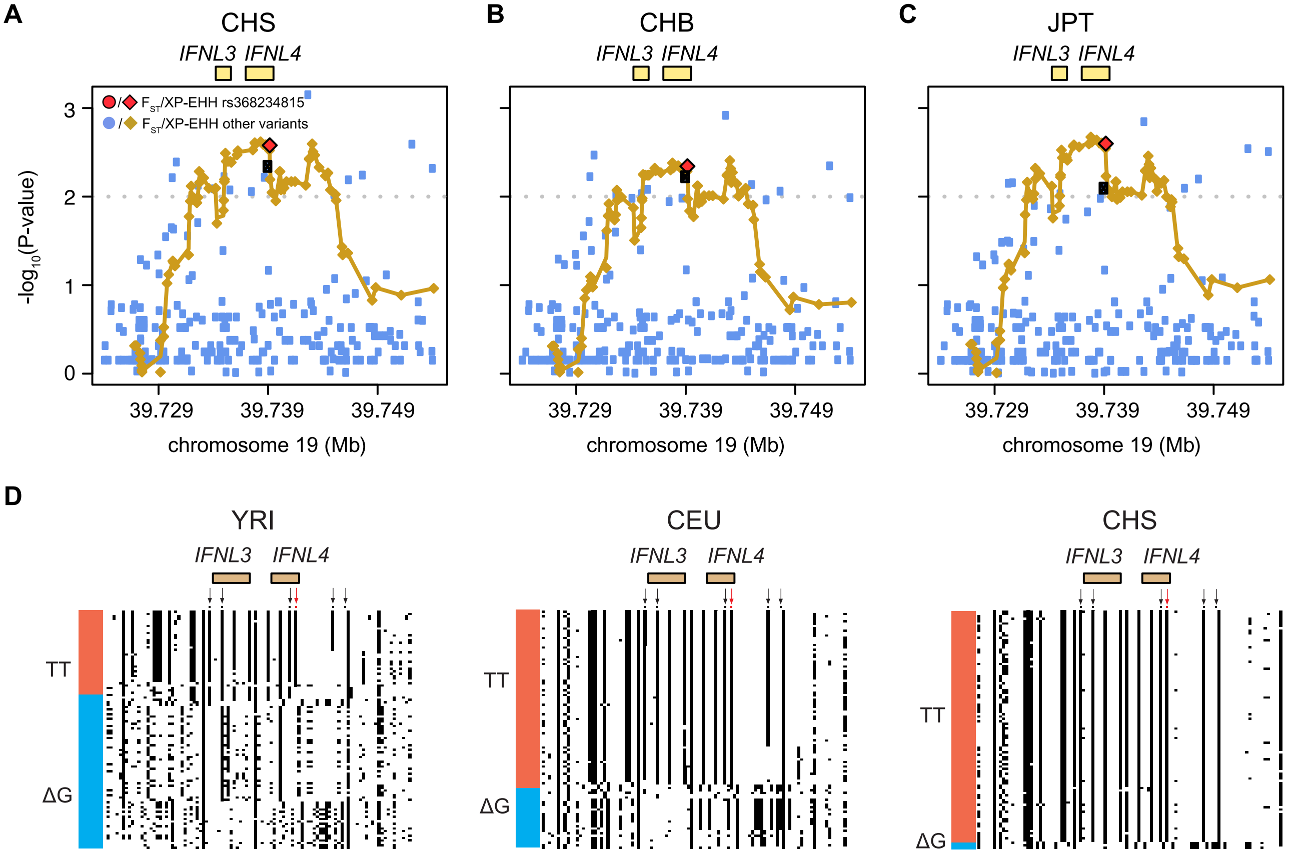

Figure 3. Empirical P-values of the F_{ST} and XP-EHH analysis (depicted as dots or diamonds, respectively) in the 30 Kb genomic locus around *IFNL4* (chr19:39724153–39754153) for (A) CHS, (B) CHB, and (C) JPT using YRI as background. All XP-EHH values are connected by a fitting curve (yellow line). The 1% tail of the genomic empirical distribution is indicated by the horizontal, dashed line. (**d**) Haplotype structure in the same region as above, for an African (YRI), European (CEU), and East Asian (CHS) population. Columns represent SNPs with a derived allele frequency >5% in at least one population (n = 99 SNPs), with the ancestral allele in white, and the derived allele in black. Lines represent the haplotypes they fall in, as inferred with SHAPEIT by the 1000 Genomes consortium [24]. Haplotypes were sorted based on rs368234815 (red arrow) and SNPs in perfect LD with it in CHS (black arrows); see also Table 2 and Figure 4. The bar on the left-hand side of each plot indicates the haplotypes that carry the TT allele (red) or the ΔG allele (blue).
doi:10.1371/journal.pgen.1004681.g003

for the SDN model is ~95% in East Asia and ~80% in Europe, corresponding to Bayes factors (Bayesian measures of relative model support [32]) of ~10 and ~4, respectively. This provides substantial and robust evidence for the SDN model, compared to the SSV and NTR models for East Asian and European populations according to Jeffrey's interpretation [33]. Therefore, we conclude that the TT allele was likely positively selected upon appearance. The ABC-based parameter estimates are less reliable than the model choice [23] because they always have large credible intervals (Bayesian measures of confidence). However, the posterior distributions have modes that differ from the modes of the prior distributions, indicating that they are determined by information from the data and not by the prior (Fig. S5). Also, the estimates are quite concordant within and between continental groups (Fig. S5, Table 3). So while they should be interpreted with appropriate caution, the estimates do provide additional useful information about the model and timing of selection. We infer that the TT allele emerged before the out-of-Africa migration (estimated t_{mut}≈55,900 years ago (41,360–68,640)) and was immediately, or shortly thereafter, targeted by moderate positive selection (selection coefficient, s_A, ≈0.58% (0.17–1.23)); we estimate that selection intensified substantially outside of Africa, with the selection strength nearly quadrupling in Europe and in Asia (s_{NA}≈2.6% (0.6–4.8); Table 3, Fig. S5).

One important aspect of the simulations is the mode of dominance (also known as the genetic model), and the ABC analysis above was performed on simulations under a perfectly additive model where heterozygotes have half the fitness effect of homozygotes (dominance coefficient h = 0.5). This model is reasonable because in TT/ΔG heterozygotes only one *IFNL4* copy is truncated, and because genetic studies show that the odds ratios (ORs) for HCV clearance in heterozygotes are intermediate to those in the two homozygotes [17]. These two arguments argue strongly against a model of complete dominance for TT as realistic, but other models are more difficult to discard *a priory*. We thus compare three dominance models: (1) a fully recessive model for the TT allele (h = 0), (2) the perfectly additive model used above (h = 0.5), and (3) a supra-additive model where the additive effect is non-linear and heterozygotes are closest in fitness to ΔG homozygotes. This model has been proposed based on the ORs for the intronic *IFNL4* variant, rs12979860 which is in high LD with rs368234815 and is thus a good proxy for the dominance effects of TT (Table 2) [34]. Based on those results we use a

Table 1. F_{ST} values and for F_{ST}, XP-EHH, iHS and Fay and Wu's H (FW) the empirical P-values are shown for rs368234815 in every population.

Population	F_{ST}	F_{ST} P-value	XP-EHH P-value	iHS P-value	FW P-value
CHS	0.63	0.005	0.003	-[b]	0.01
CHB	0.60	0.006	0.005	0.22	0.01
JPT	0.56	0.008	0.003	0.05	0.01
GBR	0.22	0.074	0.071	0.03	0.07
CEU	0.31	0.040	0.041	0.02	0.04
FIN	0.26	0.059	0.059	0.09	0.06
TSI	0.15	0.120	0.070	0.05	0.10
CLM	0.09	0.172	0.201	0.06	0.09
MXL	0.04	0.368	0.145	0.50	0.11
PUR	0.19	0.064	0.029	0.10	0.06
ASW	−0.01	0.684	0.634	0.08	0.20
LWK	0.05	0.036	0.043	0.03	0.12
YRI	-[a]	-[a]	-[a]	0.14	0.21

[a]F_{ST} and XP-EHH were not calculated for YRI, which is the background population.
[b]iHS requires a MAF of 5%, which is not given for CHS.
doi:10.1371/journal.pgen.1004681.t001

dominance coefficient h = 0.38 (see Note S4). When we compare the three dominance models in East Asia, regardless of the selection model, the fully recessive model has marginal support (4%), with the two additive models showing similar posterior probabilities (slightly higher for additive: 56%, than supra-additive: 44%, Fig. 5C and Note S4). When we compare the ABC results in the two additive models, they both strongly support the SDN model over the SSV model (95% in the additive model and 90% in the supra-additive model, corresponding to a Bayes factor of ~12), and both models provide virtually no support for the neutral model (Figure 5B–D and Note S4). Parameter estimates also agree well among these two models (Fig. S5, Fig. S6 and Note S4). Therefore our results in East Asia validate the use of an additive model and show that the ABC inferences are not sensitive to the particularities of the additive model used. In European population the results are less clear, just as in the original ABC analysis and as expected given the weaker signatures of selection. Still, these results also support the two additive models (36% support for additive and 38% for supra-additive; Fig. 5C) as well as the SDN model (~81% support for SDN in both the additive and the supra-additive, corresponding to a Bayes factor ~4, Fig. 5B and D, Note S4).

These results show a complex selection history for the TT allele, with selection starting upon appearance of the allele but with intensity changing over time and geographic range. The model is consistent with all our observations, including the marginal evidence for selection observed in non-Asian populations (Table 1). It is interesting that we infer selection on the TT allele even in Yoruba, where the signature is undetectable with classical methods likely because of weak selection and lower frequency although the TT allele shows clear signatures of homozygosity (Fig. 3D). Interestingly, and in agreement with this model, we do observe some signatures of positive selection in another African population, the Luhya. It remains possible that the advantage of the TT allele was counteracted by additional selective forces in Africa that maintained the TT allele at an intermediate frequency, such as balancing selection, although we note that the locus lacks classical signatures of long-standing balancing selection (Note S5, Table S7).

Discussion

Here we show that functional IFN-λ4 is under purifying selection throughout the mammal clade while positive selection has favored the elimination of IFN-λ4 through pseudogenization in humans. Selection on the TT allele has been particularly strong in specific populations, leading to extremely high frequency of the pseudogene and subsequent virtual loss of IFN-λ4. This event is phenotypically relevant: not only is IFN-λ4 biologically important [17,18] and evolutionary conserved, but the loss of IFN-λ4 through pseudogenization shows remarkable association with improved HCV clearance [17,19,20].

The precise reason behind the advantage of IFN-λ4 elimination is unknown, but its immunological role and clear antiviral activity against HCV [17] make exposure to pathogens (and in particular viral agents) the most likely selective force. However, due to its slow progression into fatal disease [35] HCV is unlikely to have exerted such strong selective pressure, although we cannot completely discard this possibility. Besides HCV, it has been shown that functional IFN-λ4 has antiviral activity against coronaviruses [18], while the IFN-λ4 pseudogene increases susceptibility to cytomegalovirus retinitis among HIV-infected patients [36]. Suggesting that IFN-λ4 pseudogenization is likely associated with several phenotypic traits. It is perhaps surprising that suppression of an antiviral protein results in improved viral clearance, although it has for example been shown that during chronic infection blockage of persistent signaling of IFN I (a different type of interferon) can improve viral clearance [37,38].

We showed that a complex selective regime, with variation in selection strength in different geographical areas, best explains the history of the *IFNL4* locus. Signatures of non-neutral evolution have been detected in other interferons, including at least one other *IFNL* family member (*IFNL1* or *IFNL2*) [21]. Although the mode and *tempo* of selection in these other *IFNL* genes are not well understood, together these observations suggest that IFN-λ proteins have played an important role in recent human adaptation, probably as a consequence of their role in individuals' constant fight with pathogens. It is likely, though, that only the selective history of the *IFNL4*-TT allele had a strong influence in

Table 2. Derived allele frequency (DAF), LD to rs368234815, and signatures of selection (empirical P-values for F_{ST} and XP-EHH) for other relevant SNPs across the *IFNL*-locus.

rs ID	DAF (50 ind.)		r^2 to rs368234815		XP-EHH P-value[a]		F_{ST} P-value[a]		Signal	Ref.
	CEU	CHS	CEU	CHS	CEU	CHS	CEU	CHS		
rs368234815	0.75	0.97	-	-	0.04	0	0.04	0.01	G/F	[17]
rs4803217	0.73	0.97	0.71	1	0.12	0.01	0.08	0.01	F	[72]
rs11881222	0.75	0.97	0.8	1	0.14	0.01	0.96	0.15	S	[21]
rs8103142	0.76	0.99	0.85	0.33	0.05	0	0.06	0.01	S	[21]
rs28416813	0.7	0.96	0.78	0.74	0.06	0	0.09	0.01	S	[21]
rs12979860	0.74	0.97	0.85	1	0.04	0	0.06	0.01	S/G	[6,21]
rs8109886	0.58	0.97	0.46	1	0.21	0	0.04	0	S*	
rs8099917	0.16	0.03	0.4	1	0.2	NA	0.13	0.48	G	[30]
rs8103362	0.73	0.97	0.01	0	0.71	0.24	0.68	0.06	S	[21]
rs59746524	0.87	0.94	0.01	0.04	0.77	0.28	0.54	0.24	S	[12]
rs30461	0.83	0.97	0	0	0.58	0.39	0	0	S	[21]

Included are variants which showed either a significant association with HCV clearance (G), functional effects (F), signatures of selection in a previous scan of this region, or novel strong signatures of natural selection (S). Location of each SNP in the *IFNL* locus is visualized in Figure 4.
[a]Calculated using YRI as background population.
*Selection signal detected in this study.
doi:10.1371/journal.pgen.1004681.t002

the rate of clearance of some viruses, at least HCV, across human groups.

It has been proposed that gene loss may exert an important role in evolution, including human evolution [39], and the loss of otherwise conserved regulatory elements may play a role in the acquisition of human-specific phenotypes [40]. Loss-of-function mutations show global signatures of purifying selection [41–43] and tend to carry detrimental effects [44]. A few exceptions exist, though, where truncating polymorphisms show signatures of positive or balancing selection [45–50]. Still, as with other targets of selection, most of these cases lack biological interpretation. In fact, *IFNL4* joins a small group of known genes where a striking signature of local adaptation is coupled with a clear molecular phenotype (e.g. [46,47,51]), which in this case is also associated with disease risk. As such, it contributes to our understanding of how recent human evolution has shaped genetic and phenotypic human diversity, including present-day heterogeneity in susceptibility to disease.

Materials and Methods

Molecular Evolution of *IFNL4* across species

In order to explore the level of functional constraint in *IFNL4*, we estimated the level of protein conservation in primate and non-primate mammals. Specifically, we assessed the ratio (dN/dS) of non-synonymous substitutions per non-synonymous site (dN) to synonymous substitutions per synonymous site (dS) across gene orthologs. Since purifying selection eliminates deleterious protein-coding changes, dN/dS decreases with negative selection and increases with relaxed constraint and positive selection.

We used human *IFNL4* reference sequence NM_001276254.2 to BLAT genomes of other species and generate multiple-species sequence alignment of *IFNL4* coding exons 1 through 5 (Table S8). The panda-predicted *IFNL4* ortholog was subsequently used as BLAT query to extract coding exons for additional non-primate species (Table S8). Further, we sequenced *IFNL4* (exons and introns) in genomic DNA and reconstructed complete *IFNL4* cDNA sequences of chimpanzee (Genbank accession JX867772), baboon (Genbank accession KC525947) and crab-eating macaque (Genbank accession KC525948). The whole *IFNL4* genomic region is absent in mouse or rat. All discovered functional *IFNL4* sequences (Table S8) where used for a multiple-sequence alignment which was created using ClustalW [52] and annotated with Jalview [53].

The alignment was analyzed with *codeml* (part of PAML4 [54]) to test various models of selection. We estimated the overall dN/dS for the complete tree and compared likelihoods for models that allowed: i) free dN/dS for each branch (i.e., lineage heterogeneity); ii) a primate-specific dN/dS; and iii) a human-specific dN/dS. Additionally, we performed tests aimed to detect site-specific signatures of positive selection across the phylogeny (branch models): i) model 1a (neutral) vs. model 2 (positive selection); ii) model 7 (neutral) vs. model 8 (with dN/dS>1); and iii) model 8a (with dN/dS = 1) vs. model 8 (with dN/dS>1).

Human population genetic data

We analyzed genome-wide data from the 1000 Genomes release (2010/11/23; phase I) [24]. We considered (1) autosomal variants detected in the low coverage sequencing, and (2) populations with information for at least 50 unrelated individuals, which was met by 13 populations from four different continents [African ancestry: YRI (Yoruba in Ibadan, Nigeria), LWK (Luhya in Webuye, Kenya), ASW (African Ancestry in Southwest US); European ancestry: GBR (British from England and Scotland), CEU (Utah

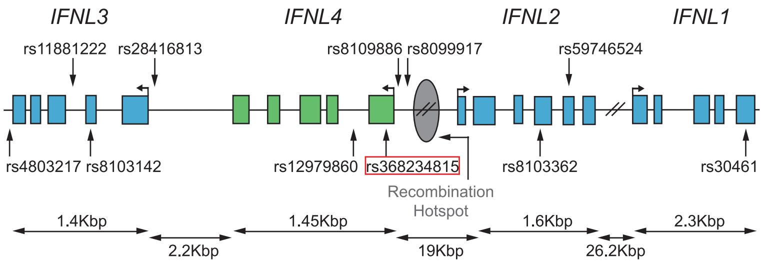

Figure 4. Map of the *IFNL* locus with locations of relevant SNPs (from Table 2) and the inferred recombination hotspot based on recombination rates from [60].
doi:10.1371/journal.pgen.1004681.g004

residents (CEPH) with Northern and Western European ancestry), FIN (Finnish from Finland), TSI (Toscani in Italia); East Asian ancestry: CHS (Han Chinese South), CHB (Han Chinese in Beijing, China), JPT (Japanese in Toyko, Japan); American ancestry: MXL (Mexican Ancestry in Los Angeles, CA), CLM (Colombian in Medellin, Colombia), PUR (Puerto Rican in Puerto Rico)]. As an exception PUR (Puerto Rico) was analyzed although it contains only 44 individuals. Some analyses were performed both by population and by continent; in these cases the continental groups contain 150 randomly selected, unrelated individuals (America 144) with an equal contribution from each population within the continent.

For the rs368234815 ΔG/TT frameshift-substitution variant the 1000 Genomes dataset only contains the T insertion/deletion variant rs11322783 (-/T, chr19:39739154, dbSNP b138), while the substitution rs74597329 (G/T, chr19:39739155, dbSNP b138) is absent. This is due to the automatic variant caller failing to correctly identify an insertion and a substitution in the same genomic position. We sequenced an amplicon containing rs368234815 in 153 individuals included both in the 1000 Genomes and HapMap sets (CEU, YRI and CHB/JPT). Sequencing confirmed the presence of only two alleles (ΔG and TT) and showed good concordance with the 1000 Genomes data between our ΔG/TT genotypes and 1000 Genomes genotypes for the overlapping insertion/deletion variant rs11322783 (4 individuals of 153 tested were discordant, providing an estimated 97.4% genotype and 98.7% allele concordance rate). This validated the use of 1000 Genomes dataset for our subsequent analyses. We used the ancestral allelic state annotated in the 1000 Genomes data, which is based on the Ensembl 59 comparative 32 species alignment [55]; only SNPs with a high-confidence ancestral inference were used, and indels were excluded due to their cryptic variation patterns [56].

Signatures of selection

We used F_{ST}, iHS and XP-EHH to explore the signatures of selection of rs368234815 TT allele. F_{ST} is a measure of population differentiation and unusually high F_{ST} can indicate population-specific positive selection that drastically increases allele frequency in the population under selection [57]. To calculate F_{ST} we used the Weir and Cockerham [25] estimator implemented in vcf-tools [58].

Positively selected alleles rapidly increase in frequency with recombination having little chance to break their association with nearby variants. If the selected allele was originally in few haplotype backgrounds and it has not reached fixation, it will be associated with extended haplotype homozygosity (EHH), a pattern that will be absent for the non-selected allele. We used two statistics to explore this expectation. First, iHS [27] measures the allele-specific decay of EHH within a population by comparing the associated EHH of ancestral and derived alleles. Second, XP-EHH [26] that detects alleles that are under selection in one population only, by comparing EHH patterns both among allelic types and across populations; as such XP-EHH has higher power to detect population-specific selection. Low frequency variants break the EHH signal, so following [59] we considered only SNPs with derived allele frequency ε 5% for XP-EHH or minor allele frequency ε 5% for iHS. Local recombination rate estimates were obtained from a combined recombination map based on HapMap data [60] from Africa, European, and Asian populations. Both statistics were standardized to a mean of zero and a standard deviation of one; for iHS, scores were then binned by frequency (1%) as previously suggested [27]. Correlation of F_{ST} with XP-EHH (CHS vs. YRI) or iHS (CHS) was calculated for all variants present in the respective dataset with Spearman's rank correlation test implemented in R [61].

We used each of these statistics to analyze every non-African population; for between-population comparisons we used Yoruba as background, unless noted otherwise. To assess the putative effects of this choice of populations we repeated the analyses for continental groups, for different background populations, and for SNPs that have their lowest allele frequency in Yoruba. In all cases the empirical P-values were obtained by comparing the score for rs368234815 to the whole-genome empirical distribution of the respective statistic. Since this is a hypothesis-driven analysis with a single variant analyzed within a single locus, no multiple testing or genome-wide corrections are needed.

We also applied tests that analyze the signatures of selection in the *IFNL4* genetic region (~2.5 kb). Here we show results for Fay and Wu's H test [28], which detects the excess of high-frequency derived alleles expected after a recent sweep with recombination. Significance was estimated using 10,000 standard neutral coalescent simulations [62]. Because demography affects the SFS and can cause spurious results if not properly accounted for, our simulations are run under a demographic model which includes inferred parameters for populations of African [63], European [63], Asian [63] and American [64] ancestry. A custom made perl program (Neutrality Test Pipeline) was used to calculate the statistic and corresponding P-value.

ABC analysis

To infer the model of selection that best fits *IFNL4* data and estimate the timing and selection strength of the TT allele, we used an Approximate Bayesian Computation (ABC) approach [22]. In particular, we followed a published approach [23], which has been previously shown to discriminate well between SDN, SSV and neutrality (NTR) [23]. In brief, this approach is based on performing a large number of simulations under different selection

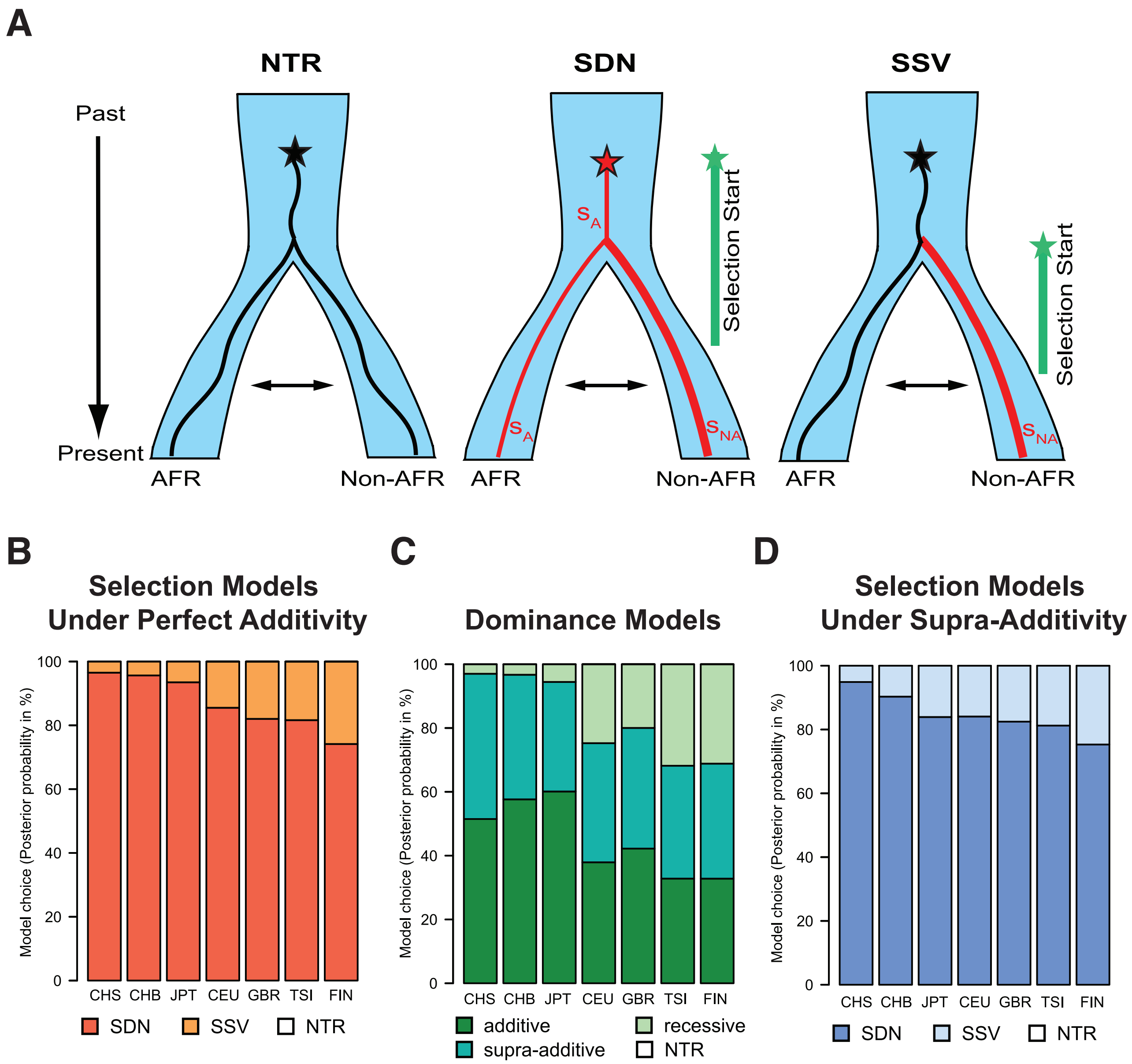

Figure 5. (A) Graphical representation of the different models of selection tested in the ABC analysis (NTR - neutral, SDN - selection on a de novo mutation, and SSV - selection on standing variation). We simulated one ancestral population that splits at the out-of-Africa event (at 51,000 years ago) into the African (AFR) and the non-African (non-AFR) populations, which experience subsequent migration. The star indicates the appearance of the focal mutation. In the first case the neutral (black) mutation appeared and evolved under neutrality in both populations. In the SDN model the advantageous mutation (red) is immediately under positive selection with strength s_A, and time when selection started t_{mut} (the prior parameter space for t_{mut} is indicated by a green line); selection strength is allowed to change in the non-African population to s_{NA}. In the SSV model the neutral (black) mutation appeared and evolved under neutrality, becoming advantageous in the non-African population (red line) at time t_{mut}. Prior parameter spaces can be found in methods. (**B**) Posterior probabilities of the model choice for the different selection models under perfect additivity. (**C**) Posterior probabilities of the model choice for the different dominance models (and neutrality, NTR). For all models except NTR the posterior probability represent the sum for the SDN and SSV selection models. (**D**) Posterior probabilities of the model choice for the different selection models under the supra-additive model. In (**B**), (**C**), and (**D**), NTR has negligible posterior probability and is therefore not visible.
doi:10.1371/journal.pgen.1004681.g005

models, with random parameters drawn from some probability distribution (called the prior distribution). Real data and simulations are compared based on summary statistics, and through a rejection scheme the simulations that most closely resemble real data help inform inferences about the best-fitting model. The parameter values that generate these simulations are then used to obtain the posterior distribution of each parameter, whose mean and standard deviation are used to perform the parameter inferences. We extended the method to consider more than one population, since two-population statistics are most informative in our case.

Specifically, the approach uses msms [65] to simulate data, custom python scripts to calculate all summary statistics, and ABCtoolbox [66] for all ABC inferences. Under both selection

Table 3. ABC results and inferred parameter estimates for the SDN model.

Pop	P (in %)	t_{mut} (in kya)	S_A (in %)	S_{NA} (in %)
CHS	97.5	54.9 (41.1–68.6)	0.55 (0.16–1.22)	2.36 (0.57–4.75)
CHB	95.1	56.0 (41.4–68.6)	0.59 (0.17–1.21)	2.56 (0.57–4.84)
JPT	91.3	56.9 (41.7–68.6)	0.60 (0.19–1.26)	2.87 (0.59–4.84)
CEU	85.2	58.3 (41.8–69.1)	0.76 (0.14–1.37)	2.86 (0.59–4.80)
GBR	84.8	59.3 (43.8–69.1)	0.72 (0.21–1.38)	2.48 (0.59–4.73)
TSI	81.8	59.2 (43.3–69.1)	0.72 (0.20–1.38)	2.48 (0.59–4.73)
FIN	74.0	59.3 (43.5–69.1)	0.70 (0.20–1.37)	2.40 (0.59–4.68)

P: posterior probability, t_{mut}: time when selection started (based on a generation time of 25 yr), S_A: selection coefficient in Africa (YRI), S_{NA}: selection coefficient in the non-African population. The 95% confidence interval (CI) is shown in brackets. Posterior distributions shown in Figure S5 and S6.
doi:10.1371/journal.pgen.1004681.t003

models, we started with uniform priors with a range as follow (see Fig. 5A):

(1) SDN model - selection strength in Africa s_A~U(0,1.5%); selection strength in non-Africa s_{NA}~U(0.5,5%); time when selection started t_{mut}~U(40,70kya)
(2) SSV model - selection strength in non-Africa s_{NA}~U(>0,5%); frequency of the allele when selection started f_0~U(0,20%); time when selection started t_{mut}~U(21,51kya)
(3) NTR model - time when mutation appears t~U(40,70kya)

Because simulations with the selected allele fixed are likely to be very different from the observed data, we conditioned on the selected allele segregating in both populations. This resulted in non-uniform prior distributions presented in Figure S5 and S6. We used 10^4 simulations to distinguish between the neutral model and the two selection models, and a larger set of 8×10^5 simulations for the more subtle distinction between the two selection models and for parameter estimation. For the simulations, we used the population history model estimated by Gravel et al. [63] and assumed a constant recombination rate of 1.76 cm/Mb throughout the region (average recombination rate in the *IFNL* locus [60]), and a perfectly additive model of dominance (h = 0.5). Lack of an appropriate demographic model for American and non-Yoruba African populations precludes analysis for those populations. The following single-population statistics were calculated: the average number of pairwise differences π, Watterson's θ, Fay and Wu's H [28] and Tajima's D [67], all for both 4 kb around the site and a 8 kb (6 kb upstream and 2 kb downstream of the site) interval around the TT allele. The between-population statistics employed were: F_{ST} [68] for the selected site, F_{ST} in 4 kb around the site, F_{ST} for the whole region, and XP-EHH on the selected site [26]. In addition, we also included the frequency of the selected allele in both populations. This resulted in a set of 16 summary statistics, which, following Wegmann et al. [69] and Peter et al. [23], was reduced to seven summary statistics using PLS-DA [70] for model choice and regular PLS for parameter inference [71]. Performance of the ABC model choice and parameter distribution for the SDN model has been assessed for each particular model (Note S3). Confidence in the choice of selection models has been supported with Bayes factors.

In addition, we investigated the influence of the dominance model in our inferences. We analyzed a recessive model for TT (h = 0), the perfectly additive model above (h = 0.5), and a supra-additive model (h = 0.38), using 500,000 simulations for each model. We run an ABC analysis for model selection with all simulations (from all three dominance models and the three selection models NTR, SDN, and SSV). We then assess the posterior probability of each dominance model regardless of selection model, and the posterior probability (and parameter estimates) of each selection model for the additive and supra-additive dominance models (see Note S4).

URLs

1000 Genomes, ftp://ftp.1000genomes.ebi.ac.uk/vol1/ftp/phase1/; GENCODE, http://pseudogene.org/psidr/; HapMap, http://hapmap.ncbi.nlm.nih.gov; XP-EHH and iHS executables, http://hgdp.uchicago.edu/Software/; VCFtools, http://vcftools.sourceforge.net; ABCtoolbox: http://www.cmpg.iee.unibe.ch/content/softwares__services/computer_programs/abctoolbox/index_eng.html; msms: http://www.mabs.at/ewing/msms/

Supporting Information

Figure S1 Empirical P-values of the F_{ST} and XP-EHH analyses for all non-Asian. populations using YRI as background within a +−15 kb region around rs368234815.
(PDF)

Figure S2 Empirical P-values of the XP-EHH and F_{ST} analysis in the *IFNL* cluster for all populations.
(PDF)

Figure S3 Haplotype structure +−15 Kb around rs368234815 for three population per continent.
(PDF)

Figure S4 Haplotype network for *IFNL4*.
(PDF)

Figure S5 Parameter estimates for the additive model using a (a) SDN or (b) SSV model of selection for Asian and European populations.
(PDF)

Figure S6 Parameter estimates for the supra-additive model using a (a) SDN or (b) SSV model of selection for Asian and European populations.
(PDF)

Figure S7 Linkage disequilibrium (LD) patterns across the *IFNL* locus in three representative populations ((a) YRI, (b) CEU, (c) CHB).
(PDF)

Note S1 Assessment of selection on other variants in the *IFNL-locus*.
(PDF)

Note S2 Geographical origin of the rs368234815 TT variant.
(PDF)

Note S3 Performance of the ABC approach.
(PDF)

Note S4 Investigation of the effects of different dominance models in the ABC analysis.
(PDF)

Note S5 Investigation of signatures of balancing selection in Africa, and of positive selection for non-synonymous variants on ΔG background.
(PDF)

Table S1 PAML results using either (a) all species or (b) only primates.
(PDF)

Table S2 Frequency of rs368234815 TT allele in the 1000 Genomes dataset and the subset of 50 unrelated individuals per population used for analyses.
(PDF)

Table S3 F_{ST} values and corresponding empirical P-values for rs368234815 using different background populations: (a) ASW, (b) LWK (c) GBR, and (d) in the continental comparison. Table (e) shows the empirical P-value of F_{ST} for rs368234815 based on genome-wide SNPs with lowest frequency in YRI (compared to ASW and LWK).
(PDF)

Table S4 Empirical P-values for the XP-EHH analysis for rs368234815 using (a) GBR as background population or (b) in continental comparison.
(PDF)

Table S5 Diversity associated with the TT haplotype in each population, as measured with Watterson's estimator and using only TT/TT homozygous individuals.
(PDF)

Table S6 ABC results and inferred parameter estimates for (a) the SSV model and (b) the neutral model.
(PDF)

Table S7 Tajima's D (TD), HKA, and MWUhigh results.
(PDF)

Table S8 *IFNL4* orthologous exons retrieved through BLAT search with human reference *IFNL4*-ΔG (for primate species) and the panda ortholog (for non-primate species).
(PDF)

Acknowledgments

We thank Cesare de Fillipo, João Teixeira, Bárbara Bitarello, Anna B. Stittrich and Montgomery Slatkin for helpful discussions and/or comments on the manuscript.

Author Contributions

Conceived and designed the experiments: AMA LPO. Contributed reagents/materials/analysis tools: WT LPO. Wrote the manuscript: AMA FMK. Designed the study and performed the population genetics analyses: FMK AMA. Performed the comparative genomics analysis: MYD. Performed the ABC analyses: BP EHS FMK RN. Generated data: WT LPO. Supervised the project: AMA. Assisted in the writing of the manuscript: BP MYD EHS WT LPO RN.

References

1. Donnelly RP, Kotenko SV (2010) Interferon-Lambda: A New Addition to an Old Family. Journal of Interferon & Cytokine Research 30: 555–564.
2. Sheppard P, Kindsvogel W, Xu W, Henderson K, Schlutsmeyer S, et al (2002) IL-28, IL-29 and their class II cytokine receptor IL-28R. Nat Immunol 4: 63–68.
3. Kotenko SV, Gallagher G, Baurin VV, Lewis-Antes A, Shen M, et al (2002) IFN-θs mediate antiviral protection through a distinct class II cytokine receptor complex. Nat Immunol 4: 69–77.
4. Tanaka Y, Nishida N, Sugiyama M, Kurosaki M, Matsuura K, et al (2009) Genome-wide association of IL28B with response to pegylated interferon-α and ribavirin therapy for chronic hepatitis C. Nat Genet 41: 1105–1109.
5. Thomas DL, Thio CL, Martin MP, Qi Y, Ge D, et al (2009) Genetic variation in IL28B and spontaneous clearance of hepatitis C virus. Nature 461: 798–801.
6. Ge D, Fellay J, Thompson AJ, Simon JS, Shianna KV, et al (2009) Genetic variation in IL28B predicts hepatitis C treatment-induced viral clearance. Nature 461: 399–401.
7. Hanafiah KM, Groeger J, Flaxman AD, Wiersma ST (2013) Global epidemiology of hepatitis C virus infection: new estimates of age-specific antibody to HCV seroprevalence. Hepatology 57: 1333–1342.
8. Perz JF, Armstrong GL, Farrington LA, Hutin YJ, Bell BP (2006) The contributions of hepatitis B virus and hepatitis C virus infections to cirrhosis and primary liver cancer worldwide. J Hepatol 45: 529–538.
9. Dill MT, Duong FH, Vogt JE, Bibert S, Bochud P, et al (2011) Interferon-Induced Gene Expression Is a Stronger Predictor of Treatment Response Than {IL28B} Genotype in Patients With Hepatitis C. Gastroenterology 140: 1021–1031.e10.
10. Fukuhara T, Taketomi A, Motomura T, Okano S, Ninomiya A, et al (2010) Variants in {IL28B} in Liver Recipients and Donors Correlate With Response to Peg-Interferon and Ribavirin Therapy for Recurrent Hepatitis C. Gastroenterology 139: 1577–1585.e3.
11. Langhans B, Kupfer B, Braunschweiger I, Arndt S, Schulte W, et al (2011) Interferon-lambda serum levels in hepatitis C. J Hepatol 54: 859–865.
12. Sugiyama M, Tanaka Y, Wakita T, Nakanishi M, Mizokami M (2011) Genetic variation of the IL-28B promoter affecting gene expression. PLoS One 6: e26620.
13. Honda M, Sakai A, Yamashita T, Nakamoto Y, Mizukoshi E, et al (2010) Hepatic {ISG} Expression Is Associated With Genetic Variation in Interleukin 28B and the Outcome of {IFN} Therapy for Chronic Hepatitis C. Gastroenterology 139: 499–509.
14. Urban TJ, Thompson AJ, Bradrick SS, Fellay J, Schuppan D, et al (2010) IL28B genotype is associated with differential expression of intrahepatic interferon-stimulated genes in patients with chronic hepatitis C. Hepatology 52: 1888–1896.
15. de Castellarnau M, Aparicio E, Parera M, Franco S, Tural C, et al (2012) Deciphering the interleukin 28B variants that better predict response to pegylated interferon-α and ribavirin therapy in HCV/HIV-1 coinfected patients. PLoS One 7: e31016.
16. Smith KR, Suppiah V, O'Connor K, Berg T, Weltman M, et al (2011) Identification of improved IL28B SNPs and haplotypes for prediction of drug response in treatment of hepatitis C using massively parallel sequencing in a cross-sectional European cohort. Genome medicine 3: 57.
17. Prokunina-Olsson L, Muchmore B, Tang W, Pfeiffer RM, Park H, et al (2013) A variant upstream of IFNL3 (IL28B) creating a new interferon gene IFNL4 is associated with impaired clearance of hepatitis C virus. Nat Genet 45: 164.
18. Hamming OJ, Terczyńska-Dyla E, Vieyres G, Dijkman R, Jørgensen SE, et al (2013) Interferon lambda 4 signals via the IFNλ receptor to regulate antiviral activity against HCV and coronaviruses. EMBO J.
19. Aka P, Kuniholm MH, Pfeiffer RM, Wang AS, Tang W, et al (2013) Association of the IFNL4-ΔG Allele with Impaired Spontaneous Clearance of Hepatitis C Virus. Journal of Infectious Diseases 32: 3055–65. doi: 10.1038/emboj.2013.232.
20. Bibert S, Roger T, Calandra T, Bochud M, Cerny A, et al (2013) IL28B expression depends on a novel TT/-G polymorphism which improves HCV clearance prediction. J Exp Med 210: 1109–1116.
21. Manry J, Laval G, Patin E, Fornarino S, Itan Y, et al (2011) Evolutionary genetic dissection of human interferons. J Exp Med 208: 2747–2759.
22. Beaumont MA, Zhang W, Balding DJ (2002) Approximate Bayesian computation in population genetics. Genetics 162: 2025–2035.
23. Peter B, Huerta-Sanchez E, Nielsen R (2012) Distinguishing between Selective Sweeps from Standing Variation and from a *De Novo* Mutation. PLoS Genet 8: e1003011.
24. McVean GA, Altshuler (Co-Chair) DM, Durbin (Co-Chair) RM, Abecasis GR, Bentley DR, et al (2012) An integrated map of genetic variation from 1,092 human genomes. Nature 491: 56–65.

25. Weir S, Cockerham CC (1984) Estimating F-Statistics for the Analysis of Population Structure. Evolution 38: 1358.
26. Sabeti PC, Varilly P, Fry B, Lohmueller J, Hostetter E, et al (2007) Genome-wide detection and characterization of positive selection in human populations. Nature 449: 913–918.
27. Voight BFAK, Wen SA, Pritchard XA, K J (2006) A Map of Recent Positive Selection in the Human Genome. PLoS Biol 4: e72.
28. Fay JC, Wu CI (2000) Hitchhiking under positive Darwinian selection. Genetics 155: 1405.
29. di Iulio J, Ciuffi A, Fitzmaurice K, Kelleher D, Rotger M, et al (2011) Estimating the net contribution of interleukin-28B variation to spontaneous hepatitis C virus clearance. Hepatology 53: 1446–1454.
30. Suppiah V, Moldovan M, Ahlenstiel G, Berg T, Weltman M, et al (2009) IL28B is associated with response to chronic hepatitis C interferon-α and ribavirin therapy. Nat Genet 41: 1100–1104.
31. Fu W, Akey JM (2013) Selection and Adaptation in the Human Genome. Annu Rev Genomics Hum Genet 14:467–89. doi: 10.1146/annurev-genom-091212-153509.
32. Kass RE, Raftery AE (1995) Bayes factors. Journal of the american statistical association 90: 773–795.
33. Jeffreys H (1998) The theory of probability. Oxford University Press.
34. Shebl FM, Pfeiffer RM, Buckett D, Muchmore B, Chen S, et al (2011) IL28B rs12979860 genotype and spontaneous clearance of hepatitis C virus in a multi-ethnic cohort of injection drug users: evidence for a supra-additive association. Journal of Infectious Diseases 204: 1843–1847. jir647.
35. Freeman A (2001) Estimating progression to cirrhosis in chronic hepatitis C virus infection. Hepatology 34: 809–816.
36. Bibert S, Wojtowicz A, Taffé P, Manuel O, Bernasconi E, et al (2014) The IFNL3/4 [DELTA]G variant increases susceptibility to cytomegalovirus retinitis among HIV-infected patients. AIDS: [In Press].
37. Teijaro JR, Ng C, Lee AM, Sullivan BM, Sheehan KCF, et al (2013) Persistent LCMV Infection Is Controlled by Blockade of Type I Interferon Signaling. Science 340: 207–211.
38. Wilson EB, Yamada DH, Elsaesser H, Herskovitz J, Deng J, et al (2013) Blockade of Chronic Type I Interferon Signaling to Control Persistent LCMV Infection. Science 340: 202–207.
39. Olson MV (1999) When less is more: gene loss as an engine of evolutionary change. Am J Hum Genet 64: 18.
40. McLean CY, Reno PL, Pollen AA, Bassan AI, Capellini TD, et al (2011) Human-specific loss of regulatory DNA and the evolution of human-specific traits. Nature 471: 216.
41. MacArthur DG, Balasubramanian S, Frankish A, Huang N, Morris J, et al (2012) A Systematic Survey of Loss-of-Function Variants in Human Protein-Coding Genes. Science 335: 823–828.
42. Montgomery SB, Goode D, Kvikstad E, Albers CA, Zhang Z, et al (2013) The origin, evolution and functional impact of short insertion-deletion variants identified in 179 human genomes. Genome Res 23: 749–61. doi: 10.1101/gr.148718.112.
43. Yngvadottir B, Xue Y, Searle S, Hunt S, Delgado M, et al (2009) A Genome-wide Survey of the Prevalence and Evolutionary Forces Acting on Human Nonsense {SNPs}. Am J Hum Genet 84: 224–234.
44. Frischmeyer PA, Dietz HC (1999) Nonsense-mediated mRNA decay in health and disease. Hum Mol Genet 8: 1893–1900.
45. Andrés AM, Dennis MY, Kretzschmar WW, Cannons JL, Lee-Lin SQ, et al (2010) Balancing selection maintains a form of ERAP2 that undergoes nonsense-mediated decay and affects antigen presentation. PLoS Genet 6: e1001157.
46. Hamblin MT, Rienzo AD (2000) Detection of the Signature of Natural Selection in Humans: Evidence from the Duffy Blood Group Locus. Am J Hum Genet 66: 1669–1679.
47. Wang XAG, Zhang WEA, Jianzhi(2006) Gene Losses during Human Origins. PLoS Biol 4: e52.
48. Xue Y, Daly A, Yngvadottir B, Liu M, Coop G, et al (2006) Spread of an inactive form of caspase-12 in humans is due to recent positive selection. The American Journal of Human Genetics 78: 659–670.
49. MacArthur DG, Seto JT, Raftery JM, Quinlan KG, Huttley GA, et al (2007) Loss of ACTN3 gene function alters mouse muscle metabolism and shows evidence of positive selection in humans. Nat Genet 39: 1261–1265.
50. Seixas S, Ivanova N, Ferreira Z, Rocha J, Victor BL (2012) Loss and Gain of Function in *SERPINB11*: An Example of a Gene under Selection on Standing Variation, with Implications for Host-Pathogen Interactions. PLoS One 7: e32518.
51. Tishkoff SA, Reed FA, Ranciaro A, Voight BF, Babbitt CC, et al (2007) Convergent adaptation of human lactase persistence in Africa and Europe. Nat Genet 39: 31–40.
52. Thompson JD, Gibson T, Higgins DG (2002) Multiple sequence alignment using ClustalW and ClustalX. Current protocols in bioinformatics: Chapter 2. Unit 2.3. doi: 10.1002/0471250953.bi0203s00.
53. Waterhouse AM, Procter JB, Martin DM, Clamp M, Barton GJ (2009) Jalview Version 2—a multiple sequence alignment editor and analysis workbench. Bioinformatics 25: 1189–1191.
54. Yang Z (2007) PAML 4: phylogenetic analysis by maximum likelihood. Mol Biol Evol 24: 1586–1591.
55. Paten B, Herrero J, Fitzgerald S, Beal K, Flicek P, et al (2008) Genome-wide nucleotide-level mammalian ancestor reconstruction. Genome Res 18: 1829–1843.
56. Kvikstad EM, Duret L (2014) Strong Heterogeneity in Mutation Rate Causes Misleading Hallmarks of Natural Selection on Indel Mutations in the Human Genome. Mol Biol Evol 31: 23–36.
57. Nielsen R (2005) Molecular signatures of natural selection. Annu Rev Genet 39: 197–218.
58. Danecek P, Auton A, Abecasis G, Albers CA, Banks E, et al (2011) The variant call format and VCFtools. Bioinformatics 27: 2156–2158.
59. Grossman S, Andersen K, Shlyakhter I, Tabrizi S, Winnicki S, et al (2013) Identifying Recent Adaptations in Large-Scale Genomic Data. Cell 152: 703–713.
60. Frazer KA, Ballinger DG, Cox DR, Hinds DA, Stuve LL, et al (2007) A second generation human haplotype map of over 3.1 million SNPs. Nature 449: 851–861.
61. Team RC (2011) R: A language and environment for statistical computing. Vienna, Austria: R Foundation for Statistical Computing; 2012. Open access available at: http://cran.r-project.org
62. Hudson RR (2002) Generating samples under a Wright–Fisher neutral model of genetic variation. Bioinformatics 18: 337–338.
63. Gravel S, Henn BM, Gutenkunst RN, Indap AR, Marth GT, et al (2011) Demographic history and rare allele sharing among human populations. Proceedings of the National Academy of Sciences 108: 11983–11988.
64. Gutenkunst RNAH, Williamson RDA, Bustamante SHA, Carlos C (2009) Inferring the Joint Demographic History of Multiple Populations from Multidimensional SNP Frequency Data. PLoS Genet 5: e1000695.
65. Ewing G, Hermisson J (2010) MSMS: a coalescent simulation program including recombination, demographic structure and selection at a single locus. Bioinformatics 26: 2064–2065.
66. Wegmann D, Leuenberger C, Neuenschwander S, Excoffier L (2010) Abctoolbox: a versatile toolkit for approximate bayesian computations. BMC Bioinformatics 11: 116.
67. Tajima F (1989) Statistical method for testing the neutral mutation hypothesis by DNA polymorphism. Genetics 123: 585.
68. Reynolds J, Weir BS, Cockerham CC (1983) Estimation of the coancestry coefficient: basis for a short-term genetic distance. Genetics 105: 767–779.
69. Wegmann D, Leuenberger C, Excoffier L (2009) Efficient approximate Bayesian computation coupled with Markov chain Monte Carlo without likelihood. Genetics 182: 1207–1218.
70. Tenenhaus M (1998) La régression PLS: théorie et pratique. Editions Technip.
71. Boulesteix A-L, Strimmer K (2007) Partial least squares: A versatile tool for the analysis of high-dimensional genomic data. Brief Bioinform 8: 32–44.
72. McFarland AP, Horner SM, Jarret A, Joslyn RC, Bindewald E, et al (2013) The favorable IFNL3 genotype escapes mRNA decay mediated by AU-rich elements and hepatitis C virus–induced microRNAs. Nat Immunol 15: 72–79.
